# Hybrid splicing minigene and antisense oligonucleotides as efficient tools to determine functional protein/RNA interactions

**DOI:** 10.1038/s41598-017-17816-x

**Published:** 2017-12-14

**Authors:** Piotr Cywoniuk, Katarzyna Taylor, Łukasz J. Sznajder, Krzysztof Sobczak

**Affiliations:** 1 Department of Gene Expression, Institute of Molecular Biology and Biotechnology, Faculty of Biology, Adam Mickiewicz University, 61-614 Poznan, Poland; 2 Present Address: Center for NeuroGenetics and the Genetics Institute, Department of Molecular Genetics and Microbiology, College of Medicine, University of Florida,, Gainesville, Florida 32610-3610 USA

## Abstract

Alternative splicing is a complex process that provides a high diversity of proteins from a limited number of protein-coding genes. It is governed by multiple regulatory factors, including RNA-binding proteins (RBPs), that bind to specific RNA sequences embedded in a specific structure. The ability to predict RNA-binding regions recognized by RBPs using whole-transcriptome approaches can deliver a multitude of data, including false-positive hits. Therefore, validation of the global results is indispensable. Here, we report the development of an efficient and rapid approach based on a modular hybrid minigene combined with antisense oligonucleotides to enable verification of functional RBP-binding sites within intronic and exonic sequences of regulated pre-mRNA. This approach also provides valuable information regarding the regulatory properties of pre-mRNA, including the RNA secondary structure context. We also show that the developed approach can be used to effectively identify or better characterize the inhibitory properties of potential therapeutic agents for myotonic dystrophy, which is caused by sequestration of specific RBPs, known as *muscleblind*-like proteins, by mutated RNA with expanded *CUG* repeats.

## Introduction

Alternative splicing (AS) is a co-transcriptional process that leads to a significant increase in proteome diversity^1–3^. AS leads to the formation of different mRNA variants from the same precursor transcript due to conditional inclusion or exclusion of alternative exons or their parts depending on the tissue type, developmental stage or disease conditions^4^. The process is controlled by a group of tissue-specific *trans*-elements that act as alternative splicing regulators, including *muscleblind*-like proteins (MBNLs), *CUG* triplet repeat binding, elav-like family proteins (CELFs) and RNA-binding fox homolog proteins (RBFOXs). They define the splicing pattern of regulated alternative exons by binding to specific RNA sequence/structure motifs (*cis*-elements)^5–7^. The inclusion or exclusion of alternative exons is primarily determined by the interplay of several alternative splicing regulators and other types of regulatory proteins, including splicing enhancers and silencers (i.e., heterogeneous nuclear ribonucleoproteins (hnRNPs) or serine/arginine-rich splicing factors (SRSFs))^8^.

MBNL proteins are key developmental regulators that are involved in different human diseases—e.g., myotonic dystrophy (DM)^5,9–12^ or Fuchs endothelial corneal dystrophy^13^. Interestingly, the MBNL1, MBNL2 and MBNL3 paralogs possess both redundant and unique functions in RNA processing and disease pathogenesis^14^. The best studied MBNL function is alternative splicing regulation^5,11,12,15^. All paralogs recognize RNA targets through an RNA-binding domain that contains four zinc fingers (ZF) organized into two tandems^11^. The MBNL-binding RNA motif is specified as a 5′-YGCY-3′ sequence (Y represents a pyrimidine)^16,17^ with preferentially single-stranded pyrimidines^18^. Depending on the location of the MBNL-binding motifs, whether upstream or downstream of the alternatively spliced exon, MBNLs serve either as repressors or activators of alternative exon inclusion, respectively^17,19^. A similar mode of action was previously described also for RBFOXs^20^ and CELFs^21^. Although many functional MBNL-binding motifs within pre-mRNAs (e.g., *Clcn1*, *cTNT*, *Atp2a1* and *Mbnl1*) have been well described, most remain unknown^14,22–25^.

The last decade has introduced several technologies for high-throughput analysis of protein/RNA interactions, including MBNL/RNA^21,26^, especially cross-linking and immunoprecipitation combined with deep sequencing (CLIP-seq)^27^ with subsequent modifications: individual-nucleotide resolution (iCLIP)^28^ and photoactivatable ribonucleoside-enhanced (PAR-CLIP)^29^. In general, CLIP-seq is a multistep method that starts from the physical cross-linking of RBPs with RNA sequences *in situ* using UV light. The protein/RNA complexes are subsequently purified by immunoprecipitation and molecular mass-based separation. Eventually, short RBP-bound RNA fragments are high-throughput sequenced^30,31^, followed by data filtration. The obtained overlapped reads form CLIP-seq clusters, which are considered to be RBP-bound regions. This method generates an enormous amount of data, enabling deeper insight into the proteins’ regulatory mode of action but also provides a high percentage of non-functional RNA binding sites and/or false-positive results. Thus, the potential RNA targets and RBP-binding motifs must still be verified in individual tests.

Herein, we described a new verification strategy that uses antisense oligonucleotides (AONs) and highly redundant hybrid splicing minigenes as a genetic construct carrying an alternative exon as well as interchangeable MBNL-sensitive regulatory elements within an upstream intron. The strategy is designed for the rapid, simple, reproducible, and efficient determination of functional protein/RNA interactions based on CLIP-seq or other whole transcriptome experiments. It also allows evaluation of the regulatory properties of MBNL-binding sites. Moreover, we show that the hybrid minigenes can be used to reveal and elucidate potential crosstalk between different RBPs in the regulation of alternative splicing. This method was tested for MBNL-specific RNA-binding regions but could be applicable for other RBPs. Finally, we emphasize the usefulness of hybrid minigenes to study the efficacy of potential therapeutic agents for inhibiting the interaction between MBNLs and expanded *CUG* repeats (*CUG* ^*exp*^) associated with myotonic dystrophy type 1 (DM1) development.

## Results and Discussion

### Two AON types effectively block functional MBNL/RNA interactions

**Figure 1:**
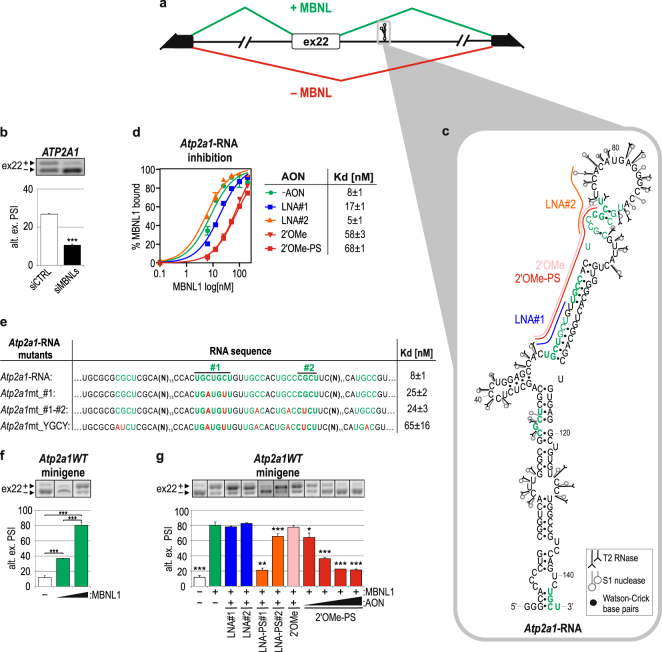
Alternative splicing of *Atp2a1* ex22 is MBNL dependent and efficiently distorted upon AON targeting of MBNL-binding regions. (**a**) A scheme illustrating the alternative splicing pattern of *Atp2a1* ex22 in the presence (green) or absence (red) of MBNLs. Black and white boxes represent constitutive and alternative exons, respectively. The RNA fragment, which was analyzed *in vitro*, comprising MBNL-binding regions #1 and #2 is marked with the gray frame. (**b**) Percentage of ex22 inclusion in *ATP2A1* endogenous mRNA upon *MBNL1,2* silencing in human cells. PSI, percent spliced in index, providing the inclusion level of an alternative exon; n = 3. (**c**) An experimentally determined secondary structure of *Atp2a1*-RNA with the YGCY motifs marked with green and in bold if present in humans and mice. Cleavages induced by RNase T2 and nuclease S1 are shown. The AONs bound *in vitro* to MBNL-binding regions #1 and #2 are accordingly indicated by blue (LNA#1), orange (LNA#2) and red lines (2′OMe/2′OMe-PS). AON, antisense oligonucleotide; 2′OMe-PS, phosphorothioated 2′-*O*-methyl; LNA, locked nucleic acid. The cleavage sites and intensities of the selected probes are shown using symbols explained in the inset. (**d**) Quantification of FBA showing the blocking properties of AONs *in vitro*. On the right, the dissociation constant (Kd) of MBNL1/*Atp2a1*-RNA complexes without (-AON) or with different AON applications; n = 4. (**e**) Quantification of FBA showing reduced MBNL1 affinity to *Atp2a1*-RNA with point mutations, especially YGCYs (marked with red). Regions #1 and #2, significant for MBNL binding, are also indicated; n = 4. (**f**) MBNL1 dose-dependent percentage of alternative ex22 inclusion in mRNA from the *Atp2a1WT* minigene in HeLa cells transfected with 200 or 500 ng of the MBNL1 expression vector per well; n = 3. (**g**) Percentage of alternative ex22 inclusion in *Atp2a1WT* mRNA upon MBNL1 overexpression and treatment with different AONs at 25–100 nM (2′OMePS) or 100 nM (others). The obtained results were compared with those of the control experiment with MBNL1 overexpression and no AON treatment (green bar); n = 3.

First, we set out to establish the most effective strategy to block protein/RNA interactions. Thus, we designed several types of AONs that target a well-defined MBNL-binding region in the *Atp2a1* transcript and tested their inhibitory properties *in vitro* and *in cellulo*. *Atp2a1* pre-mRNA contains alternative exon 22 (ex22), which as confirmed by mutagenesis, is positively regulated by the binding of all three MBNL paralogs to two YGCY motif-containing regions (region #1 and #2) that are localized within intron 22, ~110 nucleotides (nt) downstream of ex22 (Fig. 1a,c)^14,24^. We confirmed the MBNL-dependent alternative splicing of ex22 by the silencing of both *MBNL1* and *MBNL2* (Fig. 1b). Because the efficiency of MBNL binding may be modulated by the RNA’s structure^18^, we determined the secondary structure for a 145-nt-long fragment of *Atp2a1* RNA containing regions #1 and #2 through limited cleavage with two enzymatic probes that recognize single-stranded RNA^32^ (Fig. 1c, Supplementary Fig. S2a). We observed that both YGCY-rich regions were localized in the 5′-part of the semi-stable hairpin structure, surrounded by several internal loops (Fig. 1c).

To elaborate the interaction of MBNL1 with crucial YGCY motifs as well as the capacity of AONs to inhibit MBNL1 *in vitro*, we used a filter binding assay (FBA). First, we confirmed the sensitivity and specificity of this assay for a few RNA fragments that have previously been described in the literature as targets of MBNL1^24,25,33^. We did not observe any interaction of this protein with four negative control RNAs (RNA-Ctrl1-4), with or without YGCY motifs (Supplementary Fig. S1a–c). Second, we designed RNA-based AONs that selectively blocked either individual region #1 or #2 of *Atp2a1*-RNA or both simultaneously. We tested AONs with either a locked nucleic acid (LNA) or 2′-*O*-methyl (2′OMe) modification of the RNA structure with or without a phosphorothioated backbone (PS) and DNA-based AONs. The LNAs (LNA(-PS)#1 and #2) contained 10-nt residues and were complementary to individual regions #1 or #2, while the 2′OMe- and DNA-type AONs were 21-nt-long and covered both regions (2′OMe(-PS) and DNA) (Fig. 1c). Having performed the *in vitro* assay, we observed that the short LNA#2 did not prevent formation of MBNL1/RNA complexes, LNA#1 only slightly deteriorated them, and 21-nt-long AONs suppressed MBNL1/RNA complexes by approximately 8-fold (Fig. 1d, Supplementary Fig. S2b). Consistently, we observed diminished MBNL1 affinity to *Atp2a1*-RNA mutants with nucleotide substitutions in the YGCY motifs within regions #1 and #2 (Fig. 1e), confirming their previously described functional significance^24^. The lack of complete inhibition of complex formation suggests binding of MBNL1 to other YGCY motifs present within this RNA, but with significantly lower affinity. To investigate this hypothesis, we used DNA-based AONs and applied mutagenesis. We found another YGCY motif upstream of region #1 that was important for MBNL1 binding *in vitro* (Supplementary Fig. S2c–e). We showed that short LNAs were the least effective *in vitro*, most likely due to covering only a part of the motifs bound by MBNL1.

Next, we assessed the inhibitory potential of the same RNA-based AONs on MBNL-dependent alternative splicing of ex22. First, we co-transfected cells with the *Atp2a1* minigene, which encompasses a pre-mRNA sequence from exons 21 to 23 (*Atp2a1WT*), and the MBNL1-overexpressing vector. RT-PCR analysis showed substantial MBNL1-dependent enhancement of alternative ex22 inclusion (Fig. 1f,g). With the addition of different *Atp2a1*-specific AONs to this system, we observed almost complete inhibition of ex22 inclusion in LNA-PS#1- and 2′OMe-PS-treated cells as well as a significant change in LNA-PS#2. All AONs without the PS modification revealed no inhibitory effect on ex22 regulation (Fig. 1g). Moreover, the impact of 2′OMe-PS on repression of ex22 inclusion was observed even at the low 25-nM concentration (Fig. 1g). Importantly, for both LNA-PSs, but not for 2′OMe-PS, we observed a strong effect on three other MBNL-dependent alternative exons (Supplementary Fig. S2f). We expect that this effect may have been caused by LNA-PSs due to the relatively short sequence length and possible binding to a prevalent MBNL-binding motif present in many other transcripts.

In summary, we defined the structural features of the MBNL1-binding regions within *Atp2a1*-RNA. We demonstrated that the use of AONs *in vitro*, regardless of their chemistry, was an efficient tool to define the best MBNL-binding motifs. However, for *in cellulo* assays, the type of chemical modification is important. Both parameters—the length and chemical modification of 2′OMe-PS—determine its efficacy to inhibit MBNL/RNA complex formation, consistent with previously published results that showed the applicability of 2′OMe-PS AONs for blocking many different *cis*-regulatory RNA elements, including intron-exon junctions or mutation-induced alternative splice sites^34–37^. Hence, the 2′OMe-PS type was chosen for further experiments.

### AONs indicate functional MBNL-binding regions within introns but not exons

**Figure 2:**
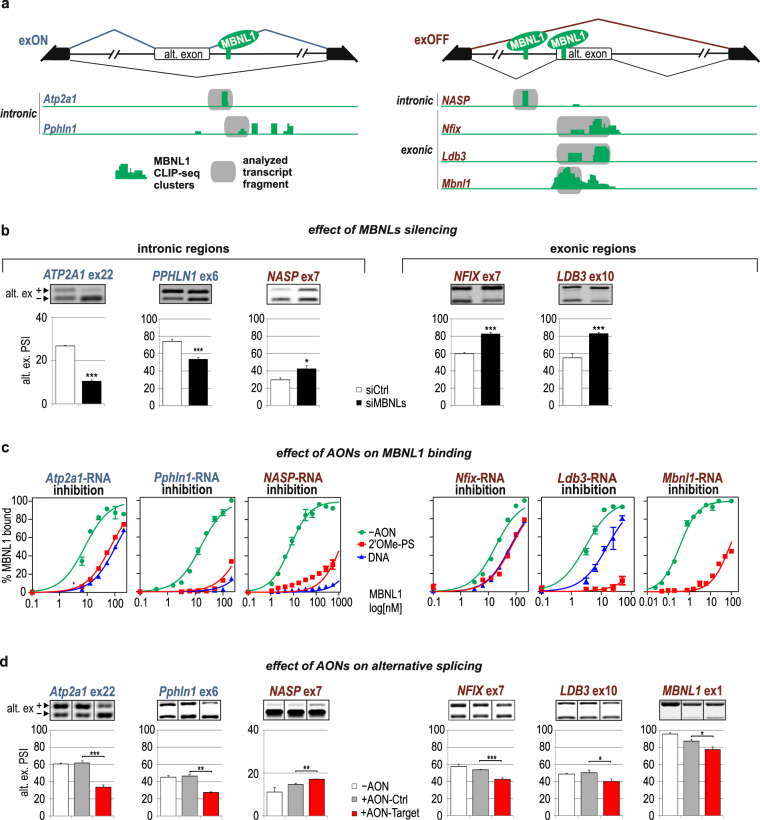
AONs indicate functional MBNL-binding regions within introns but not exons. (**a**) Schematic representation of the alternative splicing patterns of MBNL-dependent exons from *Atp2a1*, *Pphln1*, *NASP*, *Nfix*, *Ldb3* and *Mbnl1* pre-mRNAs, which depend on the localization of the MBNL-binding regions. MBNL1-specific CLIP-seq clusters are shown as green areas for each transcript. The splicing pattern for alternative exons spliced in (exON) upon MBNL binding within a downstream intron is depicted in dark blue, whereas the splicing pattern for alternative exons spliced out (exOFF) upon MBNL binding within an upstream intron or exon is depicted in burgundy. Constitutive and alternative exons are shown as black and white boxes, respectively. Intronic and exonic RNA fragments containing MBNL-binding regions targeted by AONs are marked with grey boxes. (**b**) RT-PCR analysis showing the percentage of alternative exon inclusion in the tested mRNAs in human cells after silencing of *MBNLs* and in cells treated with control siRNA (siCtrl). The results for *MBNL1* are presented in^38^; n = 3. (**c**) *In vitro* MBNL1 binding to RNA fragments derived from the studied transcripts based on FBA assays showing the blocking properties of 2′OMe-PS-AONs and DNA-AONs marked as red and blue curves, respectively; n = 2. (**d**) RT-PCR analysis showing changes in alternative splicing upon treatment with gene specific AON (AON-Target) or control AON (AON-Ctrl) in human or mouse cell lines; n = 3.

To confirm the applicability of AONs for the verification of RBP/RNA interactions, we selected five additional pre-mRNAs, besides *Atp2a1*, with potential MBNL-binding motifs within an intronic sequence downstream or upstream of *Pphln1* ex6 and *NASP ex7*, respectively, as well as within exonic sequences of ex10 of *Ldb3*, ex7 of *Nfix* and ex1 of *Mbnl1* (Fig. 2a). They were selected based on MBNL-specific CLIP-seq data deposited in MBNL Interactome Browser (MIB.amu.edu.pl)^14^. We confirmed their MBNL-dependent alternative splicing in cells upon *MBNL1,2* silencing (Fig. 2b). To date, functional MBNL-binding motifs have been well defined for three pre-mRNAs, *Atp2a1*, *Nfix* and *Mbnl1* ^19,24,38^; however, these motifs remain unknown for the other selected transcripts. We established two criteria to emerge the most probable MBNL-binding motifs in pre-mRNA: (i) the presence of an MBNL-dependent alternative exon adjacent to putative binding motifs with CLIP-seq clusters either within the exon or upstream or downstream intron up to 250 nt from the exon; (ii) overlapping of at least three YGCY sequence motifs that are at least partially conserved in human and mouse.

For each selected transcript, the identified CLIP-seq clusters covered three or more YGCY motifs, and in some instances, their number reached up to 15. First, to specify their importance in MBNL1 binding, we generated ~100- to 200-nt-long intronic (*Pphln1*-, *NASP*-) and exonic (*Nfix*-, *Ldb3*-, *Mbnl1*-) RNA fragments and designed AONs that were complementary to these regions (*Mbnl1*-RNA described in^38^) (Supplementary Fig. S3a). In FBA, we observed that certain AONs, by blocking specific RNA fragments, significantly deteriorated the MBNL1 affinity, indicating one (within *Pphln1*-, *Atp2a1*-, *NASP*-RNAs) or two (within *Nfix*-*, Ldb3*-RNA) regions composed of a string of YGCY motifs as the most essential for MBNL1/RNA interactions (Fig. 2c and Supplementary Fig. S3b). In the case of *Nfix* and *Ldb3*, for further *in cellulo* studies, we selected AONs that bound to exonic sequences and showed the strongest regulatory potential.

To confirm the functionality of the selected YGCY motifs that are essential for binding, we introduced 2′OMe-PSs into cells at 125 nM. Subsequently, we used RT-PCR to analyze the alternative splicing pattern of targeted endogenous pre-mRNAs. AONs that target intronic sequences induced significant changes in alternative exon inclusion. The *Atp2a1*- and *Pphln1*-specific 2′OMe-PSs considerably suppressed ex22 and ex6 inclusion, respectively, whereas 2′OMe-PS, which is specific for *NASP*, significantly induced inclusion of ex7 (Fig. 2d). In those transcripts, the analyzed YGCY motifs were located in downstream (*Atp2a1* and *Pphln1*) or upstream (*NASP*) introns and induced MBNL-dependent alternative exon promotion or repression, respectively. In each case, the direction and strength of alternative splicing changes upon AON application were similar to the effect of siRNA-mediated *MBNL1,2* silencing (compare Fig. 2b and d).

We next sought to identify whether targeting MBNL-binding regions with specific 2′OMe-PS in MBNL-repressed exons of *Nfix*, *Ldb3* and *Mbnl1* would also trigger the same effect as siRNA against *MBNL1,2* and lead to their enhanced inclusion. Unexpectedly, we observed the opposite effect, and found that the inclusion of ex7, ex10 and ex1, respectively, was significantly repressed by specific AONs (Fig. 2d). Silencing of *MBNL1* and 2 and AON application induced the opposite effect on the inclusion of the analyzed exons. One possible explanation for this phenomenon is that AONs, due to their interaction within the exons of their targeted pre-mRNAs, may affect the binding of other splicing modulating factors, including splicing enhancers.

We can infer that DNA oligomers are an efficient tool to screen MBNL-binding regions *in vitro* because DNA and 2′OMe-PS AONs exert comparable inhibitory properties. On the other hand, only 2′OMe-PSs indicate functional intronic MBNL-binding regions in cells. However, the strength of the AON-induced change in alternative splicing strongly depends on the primordial distribution of the splicing isoforms and potency of MBNLs to regulate particular alternative exons^39^. Moreover, we encountered difficulties in analyzing all three MBNL-binding regions within exons where AONs induced exon skipping instead of the anticipated exon inclusion.

It remains unclear what mechanism drives alternative exon regulation by MBNL proteins bound to individual RNA *cis*-elements. Two previous works proposed involvement of MBNLs in the spliceosome formation *via* interacting with U2 auxiliary factor 65 kDa subunit (U2AF65) responsible for 3′ splice site definition. Depending on the binding site localization within introns, either downstream or upstream of alternative exon, MBNLs may play a role as an enhancer for U2AF65 binding^40^ or its competitor by modifying the RNA secondary structure^41^, respectively. The other possibility is that MBNLs associated with RNA affect binding of other *trans*-factors such as CELF1^42^ and RBFOX^43^.

Analogously, the binding of MBNLs to intronic *cis*-elements tested in this study could affect U2AF65 recruitment. Therefore, AONs blocking the RNA/MBNL interaction may contribute indirectly to observed alternative splicing changes. On the other hand, binding of AON itself could directly disrupt spliceosome organization *via* RNA structure rearrangements. However, due to a relatively long distance between MBNL-binding sites and alternative exons (~110–270 nt) it seems to be less possible. Blocking intronic or exonic MBNL-binding sites by AONs might also interfere with binding of other *trans*-factors pivotal for regulation of the alternative exon inclusion. Exon skipping induced by AON targeting either the 5′ and 3′ splice sites and/or exonic splicing enhancers (ESEs) has often been reported in studies on splicing manipulation in genetic, aberrant splicing-based disorders, including Duchenne Muscular Dystrophy (DMD)^44^. Deutekom and others showed that utilizing a set of AONs that cover many regions along with an exonic sequence leads to exon exclusion^45^.

### Highly redundant hybrid *Atp2a1* minigenes confirm the functional MBNL-binding motifs in intronic and exonic sequences

**Figure 3:**
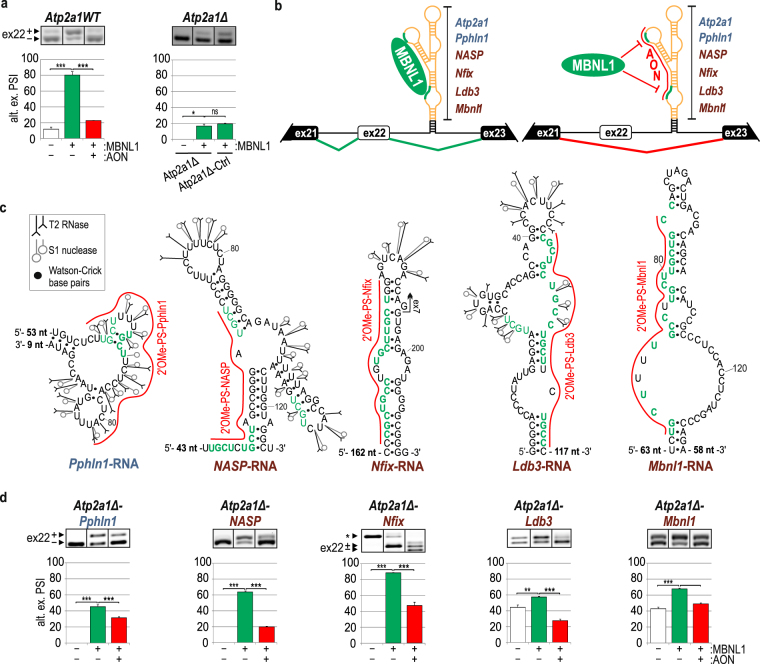
Hybrid *Atp2a1* minigenes confirm the functional intronic and exonic MBNL-binding regions. (**a**) Percentage of alternative ex22 inclusion in *Atp2a1WT* and *Atp2a1*Δ mRNA, lacking a fragment containing MBNL-binding regions, upon MBNL1 overexpression with or without 2′OMe-PS (AON) treatment; n = 3. *Atp2a1*Δ-*Ctrl*, a hybrid minigene with an inserted control sequence cassette lacking MBNL-binding regions; n = 2. (**b**) The schemes illustrate a general organization of hybrid *Atp2a1* minigenes containing an MBNL-responsive cassette derived from analyzed transcripts in the place of a natural *Atp2a1* cassette within intron 22. We expect to observe the promotion of ex22 through MBNLs binding to YGCY sequence motifs within inserted cassettes (green line) and ex22 exclusion upon AONs blocking MBNL-binding regions (red line). A thermodynamically stable structure having 14-nt-long complementary sequences derived from intron 22, restriction sites and 5-bp artificial helix, is marked with black. It is distant by 32–164 nucleotides from the MBNL-binding sites. (**c**) Experimentally determined secondary structures of selected areas of analyzed short intronic and exonic RNA fragments containing significant MBNL-binding regions complementary to specific AONs and marked with a red line. The entire structures of the analyzed *in vitro* RNA fragments are presented in Supplementary Fig. S4b. The number of not shown nucleotides is depicted on the 5′ and/or 3′ end of each structure. YGCY motifs are marked with green and in bold if present in humans and mice. *Mbnl1*-RNA was previously described^38^. (**d**) Percentage of alternative ex22 inclusion in the mRNA of a set of hybrid *Atp2a1* minigenes upon MBNL1 overexpression with or without treatment with specific 2′OMe-PS AONs. The asterisk indicates an artificial splicing isoform of the *Atp2a1Δ-Nfix* minigene; n = 3.

Previous observations led us to design hybrid minigenes as an alternative and complementary tool to test the sensitivity of RNA regulatory elements to MBNLs. To prepare the hybrid minigenes, we removed a natural 111-bp-long MBNL-binding cassette, which contains regions #1 and #2 with YGCY motifs, from intron 22 of the *Atp2a1WT* minigene (*Atp2a1Δ*). Removal of this cassette resulted in a strong reduction of the ex22 splicing response to MBNL1 overexpression (Fig. 3a). A similar effect was observed when we replaced the MBNL-binding cassette with a sequence that lacked YGCY motifs (*Atp2a1Δ*-*Ctrl*) (Fig. 3a). Subsequently, the studied RNA sequences from *Pphln1*, *NASP*, *Nfix, Ldb3* and *Mbnl1* transcripts, which contained the analyzed MBNL-binding motifs, were inserted into the *Atp2a1Δ* minigene at the site of the deleted MBNL-sensitive cassette (Fig. 3b and Supplementary Fig. S3a). The sequences were cloned in a way to preserve their autonomous RNA secondary structures and reduce the potential effect of neighboring sequences on the generated RNA structure. For that purpose, the acceptor site in *Atp2a1* minigene, which constituted a naturally stable helical region derived from intron 22 within pre-mRNA, was slightly extended by a short artificial helix which was designed in a way to not affect the secondary structure of the MBNL-binding sites (Fig. 3b). To better understand the effect of the RNA structure of the MBNL-binding regions on MBNLs activity, we experimentally determined the secondary structures of short intronic (*Pphln1*-, *NASP*-) and exonic (*Nfix*-, *Ldb3*-, *Mbnl1*-) RNA fragments representing the sequences inserted into the hybrid minigene, as described above for *Atp2a1*-RNA (Fig. 3c and Supplementary Fig. S4). RNA structure probing experiments showed that the significant MBNL-binding motifs, depicted in *in vitro* assays upon AON application, were predominantly located within semi-stable hairpin structures, either as strings of multiple YGCY motifs placed on one side of the hairpin structure with several unpaired nucleotide residues (*Ldb3*-RNA, *Mbnl1*-RNA) or within partially single-stranded regions (*Pphln1*-RNA, *NASP*-RNA). Only in *Nfix*-RNA the predicted MBNL-binding motifs were located within a stable hairpin structure with two symmetrical internal loops that were not recognized by any of the enzymatic structure probes.

Next, we co-transfected HeLa cells with one of five obtained minigenes, *Atp2a1Δ-Pphln1, Atp2a1Δ-NASP, Atp2a1Δ-Nfix, Atp2a1Δ-Ldb3* and *Atp2a1Δ-Mbnl1*, and the MBNL1 overexpressing vector. As anticipated, we observed that for all of the precursor RNAs synthesized from hybrid minigenes, inclusion of ex22 was promoted by MBNL1, similar to that for the original *Atp2a1WT* pre-mRNA (Fig. 3a,d; green bars). For two minigenes (*Atp2a1Δ-Mbnl1* and *Atp2a1Δ-Ldb3*), the endogenous MBNLs already strongly elevated ex22 inclusion compared with *Atp2a1WT* (Fig. 3d; white bars). For cells transfected with *Atp2a1Δ-Nfix*, we observed an additional artificial splicing isoform due to the presence of the remaining 5′ splice site within the cloned *Nfix* exon 7 fragment (Fig. 3d, Supplementary Fig. S4c). However, MBNL1 overexpression revealed strong inclusion of the *Atp2a1* exon 22-containing isoform (Fig. 3d).

In cells that were additionally transfected with AONs that targeted specific MBNL-binding regions, we observed a reduction of the ex22 inclusion, consistent with the effect triggered by 2′OMe-PS in *Atp2a1WT* pre-mRNA (Fig. 3a,d; red bars). In most cases, upon AON usage, the decreased level of ex22 inclusion did not reach the level observed in *Atp2a1Δ* or *Atp2a1Δ*-*Ctrl* mRNAs (Fig. 3a,d). We suppose that this effect was caused by blocking only a single MBNL-binding region with one AON, whereas the remaining motifs could still recruit MBNLs, as we observed in *in vitro* assays, and had a slight impact on ex22 inclusion.

Currently, RBP/RNA interaction studies concerning alternative splicing regulators employ a minigene preparation established on a transcript of interest containing an RBP-regulated alternative exon and RBP-binding region with its mutated variants^19,46^. Additionally, the inconvenience of the distribution of splicing isoforms is a limiting parameter. The length of the intronic sequences might also make preparations difficult^47^. To verify the high-throughput data, several of such minigenes would need to be provided, extending the time of study. Moreover, AON application is limited to intronic regulatory elements because targeting exons that are abundant in *cis*-acting regulatory sites leads to unexpected splicing misregulation.

Here, we present a new approach based on a hybrid minigene composed of an unchangeable core with an alternative exon regulated by MBNLs and exchangeable analyzed RNA regions containing potential MBNL-binding motifs combined with specific AON treatment. Of note, while we decided to use AONs, mutagenesis of potential MBNL-binding sites in RNA could be applied instead. AON application provides us with rapid and reproducible results for many intronic- and exonic-derived regulatory regions as well as the ability to evaluate their regulatory properties. Despite unexpected complications with AON usage upon potential MBNL-binding sites verification in endogenous exonic regions we decided to proceed with experiments with AONs on hybrid minigenes background as a consequence of AON application at each step of the study from *in vitro* to *in cellulo*.

Our observations clearly show that regulatory regions transferred from the original context into a different transcript may still act as *cis*-regulatory elements and regulate alternative splicing according to general principles. Our observations also indicate that MBNLs are sensitive to each of the analyzed intronic or exonic RNA fragments. Moreover, these experiments revealed that a sequence that was primarily located within an exon and acted as an ESE, when it was moved into an intronic context, became an MBNL-dependent intronic splicing enhancer (ISE). This result sheds light on the question regarding the actual mechanism of MBNL-dependent exon repression. We hypothesize that MBNLs, when bound to exonic *cis*-elements, can affect the binding or activity of other *trans*-factors that act as alternative splicing enhancers. This may explain the perplexing splicing effects that we observed when testing AONs that targeted the exonic sequences of *Nfix*, *Ldb3* and *Mbnl1* pre-mRNAs (Fig. 2d). We showed that the hybrid minigene-based approach is a useful tool to analyze MBNL-binding regulatory elements and, with great probability, that it can be successfully applied to test the functionality of other RBP-bound *cis*-elements that are naturally located in any parts of precursor or mature forms of different RNA classes. Finally, the developed hybrid minigene assay, by providing an indiscrete background, may be applicable to compare the quality of different sequences that contain MBNL-binding regions regarding the number and localization of YGCY motifs.

### Potential crosstalk between SRSF1 and MBNLs in the regulation of alternative splicing

**Figure 4:**
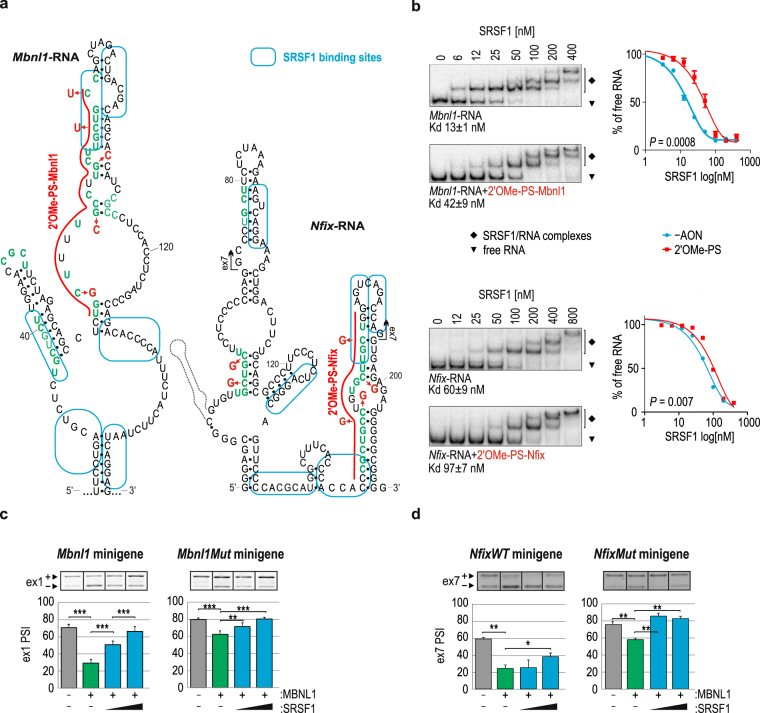
Potential crosstalk between SRSF1 and MBNLs. (**a**) Experimentally determined secondary structures of *Mbnl1*-RNA and *Nfix*-RNA with *in silico*-predicted SRSF1 binding regions marked with blue boxes^48,49^. 2′OMe-PS AONs blocking MBNL-binding regions are marked with a red line. Point mutations are marked with red. (**b**) Results of EMSA showing the interaction between SRSF1 at the indicated concentrations and 5′-^32^P-labeled *Mbnl1*-RNA (upper panel) or *Nfix*-RNA (lower panel) with either intact MBNL-binding regions or the regions blocked by individual AONs. The calculated Kd values are indicated below each electrophoretogram; n = 4. On the right is the quantification of EMSA results based on the decline of the free RNA signal in favor of forming SRSF1/RNA complexes. Antagonistic role of SRSF1 and MBNL1 in the alternative splicing regulation of (**c**) ex1 of *Mbnl1* and (**d**) ex7 of *Nfix*. Note the SRSF1 dose-dependent promotion of both exon inclusion into the mRNA of wild-type *Mbnl1* or *Nfix* (left panels) or *Mbnl1Mut* or *NfixMut* minigenes (right panels). The amount of MBNL1 or SRSF1 expressing vector constituted 500 ng or ranged from 250 to 1000 ng per well, respectively; n = 2 (*Mbnl1*), n = 3 (*Nfix*).

Blocking the exonic regions of interest with complementary AONs may lead to the unintended interruption of the general splicing machinery activity but also other *trans*-acting factors, including a large group of splicing regulators, e.g. SRSFs or hnRNPs. We decided to partially elaborate this issue. First, we attempted to find potential splicing factors that were able to bind near the MBNL-binding region using *in silico* prediction software, including *ESE finder* and *SF Map* ^48,49^. Based on these algorithms and published data^50–53^, we anticipated that the SRSF1 protein would be a putative regulatory factor, which binds close to the exonic MBNL-binding regions tested in this study and be impeded by the examined AONs (Fig. 4a). Using an *in vitro* electrophoretic mobility shift assay (EMSA), we demonstrated that recombinant SRSF1 bound to the studied exonic *Mbnl1*-RNA and *Nfix*-RNA with high affinity, ranging from 13 to 60 nM, respectively (Fig. 4b), compared with negative control RNAs (*CAG*)_20_ and (*AGG*)_20_ (Supplementary Fig. S5). The use of 2′OMe-PS-Mbnl1 and 2′OMe-PS-Nfix in *in vitro* tests significantly deteriorated the affinity by 2 to 3 fold (Fig. 4b).

To further investigate the potential mechanism of competition between SRSF1 and MBNLs *in cellulo*, we tested the alternative splicing of ex1 of the *Mbnl1* fragment within the artificial *Atp2a1* minigene pre-mRNA (*Mbnl1* minigene). Overexpression of MBNL1 led to the exclusion of ex1 (Fig. 4c), while titration of SRSF1 in the MBNL1 background progressively promoted ex1 inclusion (Fig. 4c). The results for the pre-mRNA of *Mbnl1Mut* minigene with MBNL-binding motif mutations within ex1 showed only slightly distorted activity of SRSF1 (Fig. 4c). We observed similar but weaker effects of SRSF1 activity on the alternative ex7 inclusion level in mRNA of the *NfixWT* minigene and its variant with a mutated exonic YGCY motif - *NfixMut* (Fig. 4d).

These results suggest that SRSF1 and MBNLs are antagonistic splicing regulators of ex1 of *Mbnl1* and ex7 of *Nfix* pre-mRNAs. SRSF1 very likely interacts in the vicinity of but not within MBNL-binding motifs because mutations of YGCYs do not strongly affect its activity. Potentially, binding of the tested AONs may block SRSF1 binding sterically or *via* RNA structure alterations, resulting in repression of the targeted exons. Additional studies are required to elucidate the precise mechanism and interplay between these two splicing regulators.

To date, only a few mechanisms of MBNL-mediated alternative exon regulation that implicate other *trans*-acting factors have been proposed^40–42^. Although none of them is able to explain the MBNL-mediated regulation of alternative splicing through interactions within an exonic region, we can deduce that the mode of action of MBNLs involves direct competition of two proteins for the same binding region, as described for U2AF65^41^, or indirect competition by the modulation of the RNA secondary structure and/or assistance of mediatory proteins, as shown for U2AF65^40^ and CELF1^42^. Our initial results seem to support the latter mode of action; however, this finding needs to be clarified with more data.

### The hybrid *Atp2a1* minigene is a useful tool to characterize inhibitors of adverse MBNL/RNA interactions

To emphasize the versatility of the *Atp2a1* hybrid minigene, we determined its usefulness for screening or characterizing the potency of different reagents that distort pathogenic protein/RNA interactions in myotonic dystrophy type 1 (DM1) or Fuchs endothelial corneal dystrophy.

DM1 is a neuromuscular, autosomal dominant disease that is caused by the expansion of three-nucleotide *CTG* repeats located in the 3′-untranslated region of the myotonic dystrophy protein kinase gene (*DMPK*)^54–56^. Expression of mRNA with *CUG* expansion ((*CUG*)^exp^) leads to mRNA retention in the nucleus as well as pathogenic recruitment and sequestration of nuclear proteins, including MBNLs, to an RNA hairpin-like structure formed by the repeats^55^. The longer the repeats, the more severe the missplicing of hundreds of genes due to the functional insufficiency of MBNLs^57^. On the other hand, in Fuchs endothelial corneal dystrophy, toxic (*CUG*)^exp^ RNA repeats occur within intron 2 of a transcription factor 4 pre-mRNA (*TCF4*)^13,58^.

Among the therapeutic strategies aimed at ameliorating the toxic effect of (*CUG*)^exp^, antisense oligonucleotides and small compounds have been frequently employed to specifically bind to repeats and prevent MBNL sequestration^59–62^. We have previously shown that an 8-nt-long oligonucleotide composed of LNA-PS moieties (LNA-PS-CAG-8)^63^ and erythromycin (EM)^64^ prevent MBNL sequestration on this toxic RNA by complementarily hybridizing to or potentially binding to U/U mismatches within the structure formed by (*CUG*)^exp^, respectively. These molecules diminish adverse molecular outcomes, mainly abnormalities in alternative splicing in the cell and mouse models of DM1.

**Figure 5:**
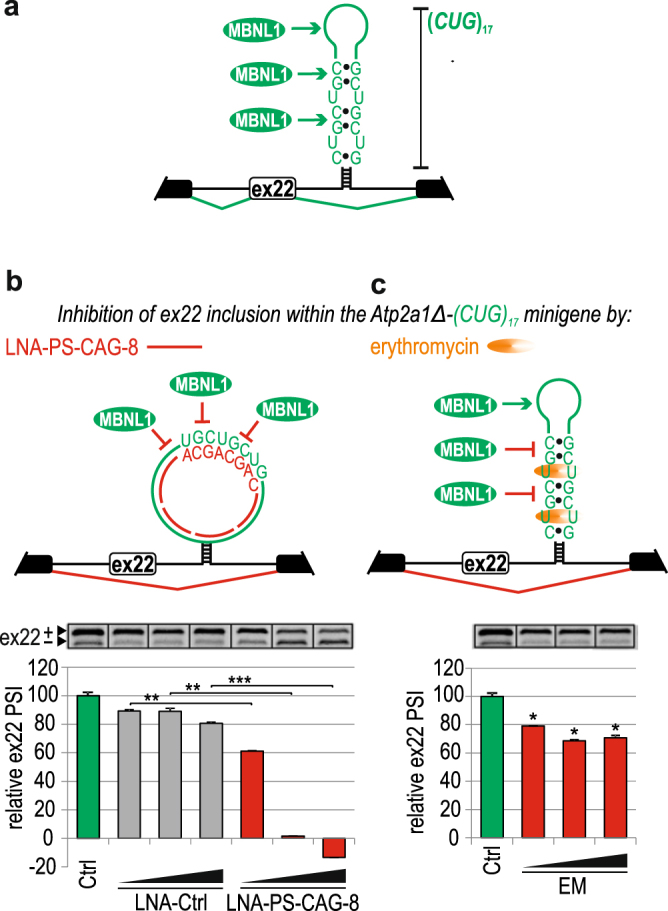
The hybrid minigene is useful to screen potential RNA-binding therapeutics. (**a**) A scheme illustrating MBNL-dependent ex22 inclusion to mRNA derived from the *Atp2a1Δ-(CUG)*_17_ minigene by extensive MBNL1 binding to the hairpin structure formed by (*CUG*)_17_. Constitutive and alternative exons are shown as black and white boxes, respectively. (**b**–**c**) *Top*; Schemes illustrating the inhibition of MBNL-dependent ex22 inclusion to mRNA derived from the *Atp2a1Δ-(CUG)*_17_ minigene by antisense oligonucleotide LNA-PS-CAG-8 (left panel) and erythromycin (EM) by their binding to the hairpin structure formed by *(CUG)*_17_ repeats. *Bottom*; reduction of alternative ex22 inclusion to *Atp2a1Δ-(CUG)*_17_ mRNA in cells treated with (**b**) LNA-PS-CAG-8 or (**c**) EM. The results are normalized to the splicing response of ex22 in *Atp2a1Δ-(CUG)*_17_ pre-mRNA without (0%) or with (100%) MBNL1 overexpression (Ctrl, green bar). The LNA-PS-CAG-8 and EM were used in a range of 25–125 nM or 50–500 µM concentrations, respectively; n = 2.

To investigate the usefulness of the hybrid minigene in the screening of potential therapeutics that target *CUG* repeats, we substituted a natural regulatory cassette of *Atp2a1* with an MBNL-binding region composed of 17 *CUG* repeats (*CUG*)_17_ (*Atp2a1Δ-(CUG)*_17_) (Fig. 5a). Our previous experiments showed that this type of sequence forms a stable RNA hairpin structure that binds all MBNLs with high affinity^14^. In this study, MBNL1 overexpression induced a strong splicing response of ex22 in the minigene’s pre-mRNA (Supplementary Fig. S6a). Additionally, we assumed that more than one MBNL1 molecule binds to the pre-mRNA of *Atp2a1Δ-(CUG)*_17_. In support of our hypothesis, hybrid minigenes expressing the pre-mRNAs containing (*CUG*)_4_ or (*CUG*)_8_ undergo MBNL-mediated splicing with lower rate of ex22 inclusion compared to *Atp2a1Δ-(CUG)*_17_ (Supplementary Fig. S6b).

Next, we chose LNA-PS-CAG-8 and EM as examples of efficient inhibitors of the MBNL/*CUG* interaction and used them in our minigene assay. Cells co-transfected with the *Atp2a1Δ-(CUG)*_17_ minigene and MBNL1 overexpressing vector were treated with different concentrations of either LNA-PS-CAG-8 or EM. We observed a strong and gradual decrease in ex22 inclusion induced by LNA-PS-CAG-8 but not by the control LNA oligonucleotide, reaching below the level established by endogenous MBNLs at a concentration of 125 nM (Fig. 5b). The EM showed a weaker effect on ex22 inclusion over a broad range of this antibiotic concentration (Fig. 5c). This result was consistent with our previous findings that showed that these two compounds have different potencies as inhibitors of the MBNL/(*CUG*)^exp^ interaction in both *in vitro* and *in vivo* models^63,64^.

These results show that the *Atp2a1Δ-(CUG)*_17_ hybrid minigene is a very sensitive tool for characterizing different MBNL/(*CUG*)^exp^ inhibitors and that after certain modifications, such as reporter gene inclusion, the hybrid minigene can be successfully applied in high-throughput screening. A related screening system, based on the detection of alternative splicing pattern changes within the *Clcn1* minigene’s pre-mRNA and including luciferase system for high-throughput analysis, has been described previously^65^. The advantage of our *Atp2a1Δ-(CUG)*_17_ minigene is its sensitivity, reproducibility and simplicity because it can be introduced into non-DM1 cell models. Moreover, the assay provides not only qualitative but also quantitative information about the potency of the studied compounds and additionally enables to monitor the differences in the number of MBNLs associated with *CUG* repeats. Investigation of potential therapeutic agents for diseases mediated by toxic RNA that disrupts the activity of RBPs, including myotonic dystrophies, is an ongoing process. Our assay could facilitate the screening of molecules on *in vitro* stages.

## Methods

### Oligonucleotides

All of the DNA sequences were purchased from oligo.pl® (Institute of Biochemistry and Biophysics PAN, Warsaw, Poland) and are listed in Supplementary Tables. All RNA-based AONs were synthesized by RiboTask™ and are listed in Table 1.

**Table 1:** RNA-based AON sequences.

AON name	Sequence 5′ → 3′^a^
**for** ***Atp2a1*** **transcript**	
LNA#1	CAGCAGCAGC
LNA#2	GUGGAAGCGG
LNA-PS#1	CAGCAGCAGC
LNA-PS#2	AAGCGGGCA
2′OMe	AGCGGGCAGUGGCAACAGCAG
2′OMe-PS	GCGGGCAGUGGCAACAGCAGC
**for other transcripts**	
2′OMe-PS-Pphln1	GGAGAAGCAAAAAGCAAGAGA
2′OMe-PS-NASP	AGCAUCCCGGCUAGCAGAGCA
2′OMe-PS-Nfix	CCAGCAAGCACAGGCAGCGGG
2′OMe-PS-Ldb3	CAGAAGCAGGCAGCAGCGGGG
2′OMe-PS-Mbnl1	GCAGCAAGCAAGGCAAAAGCA
LNA-PS-CAG-8	CAGCAGCA
LNA-Ctrl	ACGCACAAGG

^a^In each sequence, the 3′ end nucleotide was 2′OMe.

### Plasmid preparation

To obtain the *Atp2a1WT* minigene, a fragment of the *Atp2a1* gene between exons 21 and 23 containing MBNL-sensitive alternative exon 22 was amplified from mouse genomic DNA using Platinum® high fidelity *Taq* DNA polymerase (Invitrogen™) according to the manufacturer’s protocol with a SrcMres_F/SrcMres_R primer set (Ta = 55 °C) whose sequences are provided in Supplementary Table S1. These primers introduced unique restriction sites for *Xho*I and *BamH*I. To obtain the *Atp2a1Δ* minigene, a 111-bp sequence within intron 22 in the *Atp2a1WT* minigene that included the MBNL-binding regions was substituted with a 34-bp linker sequence containing unique restriction sites for *Not*I and *Sal*I. First, two fragments of the final *Atp2a1Δ* insert were amplified using the pEGFP_F/NotSal_R and NotSal_F/pEGFP_R primer sets (Ta = 60 °C) to introduce the 34-bp linker in both PCR products. Second, based on their complementarity to 34-nt linker, the obtained PCR fragments constituted the template for PCR with the pEGFP_F/pEGFP_R primer set (Ta = 60 °C) to provide a full-length *Atp2a1Δ* sequence. To obtain *Atp2a1*-hybrid minigenes, the sequences of interest from the *Pphln1*, *NASP*, *Nfix*, *Ldb3*, *Mbnl1 and Ctrl (Capzb)* genes were amplified from human (*NASP*) or mouse (others) genomic DNA with the Pph1insF/Pph1insR, NASPinsF/NASPinsR, NfixinsF/NfixinsR, Ldb3insF/Ldb3insR, Mbnl1insF/Mbnl1insR, CTRLinsF/CTRLinsR primer sets (Supplementary Table S1), respectively. The primers introduce restriction sites for *Not*I or *Sal*I. They also contained 5 additional nucleotides at the 5′ ends to introduce a hairpin structure in transcribed pre-mRNAs to stabilize the secondary structure of the fragments of interest (except for -*Mbnl1* construct). To obtain the *Atp2a1-(CUG)*_17_ minigene, oligodeoxynucleotides CTG17F and CTG17R with restriction sites for either *Not*I or *Sal*I, were used. Prior to their hybridization for one hour at RT, they were purified on a denaturing 10% polyacrylamide (PA) gel, followed by precipitation and denaturation at 95 °C for 5 min. All of the amplified DNA fragments of interest were ligated into the pEGFP-C1 vector (CloneTech) prior to transformation into DH5α bacteria cells. To obtain the *Mbnl1* minigene, a DNA sequence including the 3′ part of the intron (53 bp) and 5′ part of exon 1 (110 bp) of the *Mbnl1* gene was amplified from human genomic DNA using the Mbnl1F/Mbnl1R primer set (Ta = 55 °C), introducing restriction sites for *Not*I or *Sal*I (Supplementary Table S1). A *Mbnl1Mut* minigene was prepared in two rounds of PCR. First, two products were amplified with primers that introduced point mutations within the YGCY motifs (Mbnl1mtR/Mbnl1F as well as Mbnl1mtF/Mbnl1R; Supplementary Table S1) (Ta = 55 °C) and were purified on an agarose gel. Second, the purified PCR products were annealed and extended in 4 cycles of 92 °C for 2 min, 55 °C for 2 min, 72 °C for 2 min, and then, an Mbnl1F/Mbnl1R primer set that induced the restriction sites for *Not*I or *Sal*I was added and the reaction proceeded according to the manufacturer’s protocol at Ta = 55 °C. The obtained final PCR products, after digestion with *Not*I or *Sal*I, were ligated into the *Atp2a1Δ* minigene and were transformed into DH5α bacteria cells. The pEGFP-C1-MBNL1-41 vector for MBNL1 overexpression was previously described^38^. pEGFP-SF2 (pEGFP-C1-SRSF1) was a gift from Dr. Tom Misteli (Addgene plasmid # 17990)^66^.

### Cell culture and transfection

Human HeLa and mouse C2C12 cells were grown in high-glucose DMEM medium (Lonza) supplemented with 10% fetal bovine serum (Sigma) and 1x antibiotic/antimycotic (Sigma). Human skeletal myoblast (HSkM) cells were grown in HAM F-10 medium (Sigma) supplemented with 20% FBS, 1x antibiotic/antimycotic, 0.39 µg/ml dexamethasone (Sigma) and 10 ng/ml epidermal growth (Sigma). All cells were grown at 37 °C and in a 5% atmosphere of CO_2_. HeLa, C2C12 or HSkM cells plated in 12-well plates were transfected at 50–60% confluence with Lipofectamine® 2000 (Invitrogen™) according to the manufacturer’s protocol. Single transfection was conducted with an siRNA mix against *MBNL1* and *MBNL2* (25 nM each) (FUTURE synthesis and RiboTask™, respectively^67,68^), 50 nM AllStars negative control siRNA (Qiagen), or a specific AON at 125 nM, in which AON-Ctrl was 2′OMe-PS unspecific to a tested transcript. Co-transfection was conducted with 200 ng of the minigene and 500 ng (or as indicated in the figures) of the MBNL1, SRSF1 or GFP expressing vector. For verification of the MBNL-binding regions and inhibition of the MBNL/(*CUG*)^exp^ interaction, the co-transfection was followed by a 4-hour incubation and transfection with selected AONs. Erythromycin was added directly to the medium. The cells were harvested 48 hours after transfection.

### Splicing analyses of mature mRNA

Total RNA was isolated from cells using TRI-reagent (Sigma) according to the manufacturer’s protocol. cDNA was synthesized using GoScript™ Reverse Transcriptase (Promega) with Random Primers (Promega) according to the manufacturer’s protocol. RT-PCR was performed using GoTaq® Flexi DNA Polymerase (Promega) with the primer sets listed in Supplementary Table 1 at Ta = 55 °C. The PCR products were electrophoretically separated in 1–2% agarose gels containing 0.005% ethidium bromide, and images were captured using G:Box EF2 (Syngene). In the main figures, splicing analyses are represented by cropped sections of the original gels separated by black lines. All of the raw images of the RT-PCR analyses are shown in Supplementary Fig. 7.

### Preparation of radiolabeled RNA fragments

The templates for the transcription reactions of RNA fragments were obtained in two rounds of PCR. In the first PCR, longer products were amplified with genomic mouse or human DNA templates using specific _F/_R primer sets (the primer sequences and annealing temperatures (Ta) are listed in Supplementary Table S2). The obtained PCR products constituted a template for a second round of PCR with specific _TF/_TR primer sets harboring a promoter for polymerase T7 (Supplementary Table S2) at Ta = 55 °C. Templates for *Atp2a1*-RNA mutants were prepared in two rounds of PCR similar to that for the *Mbnl1Mut* minigene. In the first step, specific Atp2a1mt_F/Atp2a1_TR and Atp2a1mt_R/Atp2a1_TF were used. In the second round of PCR, only _TF and _TR were used. The transcription reaction was performed in 50 µl containing 10 µl of DNA template (~0.5 μg), 0.15 mM of each ribonucleotide (Invitrogen™), 0.45 mM guanosine (Sigma-Aldrich), 50 U of T7 RNA Polymerase (Ambion), 1 × T7 transcription buffer (Ambion), and 40 U of RNasin®Plus RNase Inhibitor (Promega). The transcripts were purified on a denaturing 8% PA gel (19:1 acrylamide:bisacrylamide), followed by ethanol precipitation. For 5′-end radiolabeling, 2 pmol of RNA was incubated with 2 pmol (12 mCi) of [γ-P^32^] ATP, 1 U of RNasin®Plus RNase Inhibitor (Promega) and 10 U of OPTI Kinase (Affymetrix) in 1 x reaction buffer (Affymetrix) at a total volume of 10 µl and at 37 °C for 45 min. The 5′-^32^P-labeled RNAs were subsequently run on a denaturing 8% PA gel. The bands of RNAs were visualized *via* Storage Phosphor Screen BAS-IP (IP) and a laser scanner FLA-1500 (FujiFilm) and were then excised, followed by ethanol precipitation and resuspension in 20 µl of double-distilled water.

### Chemical and enzymatic analysis of RNA structure

Analysis was conducted as previously described^38^ with slight changes. Briefly, 5′-^32^P-labeled RNA was first subjected to a denaturation and renaturation procedure in either buffer N (20 mM Tris-HCl pH 7.2, 80 mM NaCl, 2 mM MgCl_2_) for ribonucleases T1 and T2 or in a commercial S1 buffer (Fermentas) for endonuclease S1. Limited RNA digestion was initiated by mixing 5 µl of the RNA sample (~0.5 pmol) with 5 µl of a probe solution containing S1 and T2 at the concentrations indicated in Supplementary Figs S2a and S4a. An equal volume of stop solution was added to stop all reactions. The products of cleavage were separated on a denaturing 10% PA gel along with the nucleotide ladders constituting products of alkaline hydrolysis (F), lead ion cleavage (Pb) and T1 nuclease digestion (T1L). The F ladder was generated by RNA cleavage in formamide at 99 °C for 10–15 min. The T1L ladder was generated by RNA cleavage in 50 mM sodium citrate pH 4.3 and 7 M urea by addition of 10 or 15 U/µl of T1 at 55 °C for 10 min. The Pb ladder was generated by RNA cleavage in buffer N by 0.25 mM of Pb^2+^ at 55 °C for 2.5 min. The bands of RNAs were visualized *via* IP and the laser scanner FLA-1500 after overnight (O/N) exposition. *In silico* prediction of the secondary structure was performed using the *mfold* program with experimentally defined constraints.

### Recombinant proteins

The GST fusion construct with a truncated 41-kDa MBNL1 isoform sequence encoded by exons 1–4 of the MBNL1 gene with a His6 tag at the C-terminus was prepared as previously described^69^.

To generate the construct for recombinant SRSF1, 558 bp of the SRSF1 coding sequence was amplified using the pEGFP-SF2 vector (Addgene) as a template and SRSF1_F/ SRSF1_R primer set (Supplementary Table S2) and was cloned into the pRSF-Duet vector using *BamH*I and *Not*I. Sequencing of the obtained construct was followed by transformation of the *E. coli* BL21(DE3)pLysS strain (Novagen). Production of 23-kDa SRSF1 containing a His6 tag at the C-terminus was performed as previously described^70^.

### Quantification of the MBNL1/RNA interaction and its inhibition by AONs *in vitro*

The filter binding assay (FBA) was performed to assess the affinity of MBNL1 to different RNAs. 5′-^32^P-labeled RNAs (0.05 nM) were subjected to a denaturation and renaturation procedure by heating the samples at 95 °C for 1 min followed by a 10-min incubation at RT. Subsequently, the RNAs were incubated with the indicated protein concentrations (ranging from 0 to 200 nM) in buffer FB (250 mM NaCl, 15 mM KCl, 50 mM Tris-HCl pH 8.0, 0.05% Tween-20, 1 mM MgCl_2_) at 37 °C for 15 min. Studying the inhibitory property of AONs, 0.05 nM of 5′-^32^P-labeled RNAs mixed with 0.5 µM of RNA-based AONs or 20 µM of DNA-AONs (Supplementary Table S3) were subjected to the denaturation and renaturation procedure, followed by incubation at 37 °C for 15 min. Next, the indicated concentrations of MBNL1 (ranging from 0 to 200 nM) were added to each sample, followed by incubation at 37 °C for 15 min. Next, 25 µl of each sample was loaded onto the filter binding apparatus with nitrocellulose (Protran BA 85, Whatman®) and nylon (Hybond™ N + , Amersham) membranes that were previously wetted in buffer FB. The signal from the membranes was visualized *via* IP and laser scanner FLA-1500 after O/N exposition. The dissociation constant (Kd) of the MBNL1/RNA complexes treated or non-treated with different AONs was calculated in the GraphPad program using the following equation: one site specific binding curve (Y = Bmax*X/(Kd + X)).

### Quantification of the SRSF1/RNA interaction and its inhibition by AONs *in vitro*

The electrophoretic mobility shift assay (EMSA) was carried out by incubation of 0.05 nM of 5′-^32^P-labeled RNA fragments with the indicated SRSF1 concentrations (ranging from 0 to 800 nM). The reactions were performed in a volume of 10 µl in buffer A (100 mM NaCl, 20 mM HEPES-Na pH 8.0, 5% glycerol, 0.5 mM DTT, 0.2 mM EDTA, 50 µg/ml BSA (Invitrogen™)) at 37 °C for 20 min. To identify SRSF1-binding regions, 0.5 µM of 2′OMe-PS-Mbnl1 or -Nfix along with 0.05 nM of 5′-^32^P-labeled RNAs were subjected to denaturation and renaturation, followed by incubation at 37 °C for 20 min in buffer A. The samples were mixed with loading buffer (30% glycerol, 0.25% of bromophenol blue and xylene cyanol) and run on a non-denaturing 8% PA gel. The gel was subsequently dried, and the signal was visualized *via* IP and the laser scanner FLA-1500 after O/N exposition. The Kd of the SRSF1/RNA interaction was calculated based on the signal of free RNA and using the equation: one phase decay curve equation (Y = (Y0 − Plateau)*exp(−K*X) + Plateau) in Graph Pad program.

### Statistical analysis

Group data obtained *in vitro* and *in cellulo* are expressed as the means ± standard deviation (SD). The statistical significance of the RT-PCR and EMSA results was determined by two-tailed Student’s t-test using Microsoft Excel (*P < 0.05; **P < 0.01 and ***P < 0.001). Statistical analysis was calculated using 2–4 biological repeats. Similar results were obtained from at least two independent experiments.

### Data availability

All data generated or analyzed during this study are included in this published article (and its Supplementary Information files).

## Electronic supplementary material

Supplementary Information
