# Supplementary material for: Hybrid splicing minigene and antisense oligonucleotides as efficient tools to determine functional protein/RNA interactions
Source: Sci Rep. 2017 Dec 14;7:17587. doi: 10.1038/s41598-017-17816-x (PMC5730568; doi:10.1038/s41598-017-17816-x)
Supplement: Supplementary file 1 — Supplementary Information [file 41598_2017_17816_MOESM1_ESM.pdf]

SUPPLEMENTARY DATA

Hybrid splicing minigene and antisense oligonucleotides as efficient tools to determine functional protein/RNA interactions

Piotr Cywoniuk[1,#], Katarzyna Taylor[1,#], Łukasz J. Sznajder[1,2], Krzysztof Sobczak[1,*]

[1] Department of Gene Expression, Institute of Molecular Biology and Biotechnology, Faculty of Biology, Adam Mickiewicz University, Umultowska 89, 61-614 Poznan, Poland

[2] Current address Center for NeuroGenetics, Department of Molecular Genetics and Microbiology, University of Florida College of Medicine, 2033 Mowry Road PO Box 103610, Gainesville, FL 32610-3610

[#] These authors contributed equally to this work.

* Correspondence should be addressed to K.S. (ksobczak@amu.edu.pl).

Supplementary Materials & Methods

Supplementary Table 1. Sequences of primers for *in cellulo* analysis

Supplementary Table 2. Sequences of primers for *in vitro* analysis

Supplementary Table 3. Sequences of DNA antisense oligonucleotides

Supplementary Table 4. Coordinates of analyzed alternative exons in human (hg19) and mouse (mm10) genomes

Supplementary Figures

Supplementary Figure S1. The presence of YGCY motifs is not sufficient for MBNL1 binding in *in vitro* tests**.**

Supplementary Figure S2. Indication of significant MBNL1-binding motifs within *Atp2a1*-RNA *via* DNA-AONs and mutagenesis.

Supplementary Figure S3. DNA-AONs sufficiently block MBNL1-binding regions *in vitro*.

Supplementary Figure S4. Enzymatic probing of short intronic and exonic RNA fragments.

Supplementary Figure S5. Specificity of SRSF1 binding *in vitro*.

Supplementary Figure S6. Splicing sensitivity of *Atp2a1-(CUG)*$_{17}$ pre-mRNA to MBNL1 overexpression.

Supplementary Figure S7. Raw images of RT-PCR analyses of alternative splicing.

Supplementary Materials & Methods

Supplementary Table S1

Primers' sequences for *in cellulo* analysis

**Minigenes' preparation**		
**Primer name**	**Sequence 5'→3'**	
SrcMres_F	ACTACTCGAGGATCTTCAAGCTCCGGGC	
SrcMres_R	ACTGGATCCAGCAATCAGCTAGTCAGTT	
SrcMins_F	GATCTTCAAGCTCCGGGCCCTG	
SrcMins_R	AGCAATCAGCTAGTCAGTTGCC	
pEGFP_F	AAAGACCCCAACGAGAAGCGCGATC	
pEGFP_R	CCTCTACAAATGTGGTATGGCTGATTATGATCAG	
NotSal_F	GCGGCCGCTAAGTCCAGTAGAGCTACTAGTCGACTGGCCGCAGGCTGCTGGGCTGG	
NotSal_R	GTCGACTAGTAGCTCTACTGGACTTAGCGGCCGCTGACCGCAGGCTGCGCATGGGCT	
Mbnl1insF	ACTGCGGCCGCTTTCCTGACGTCTCTGCTGC	
Mbnl1insR	ATCGTCGACTCTCCTGATTAGAAGTAGAAATGG	
NfixinsF	ACTGCGGCCGCAGGACGTTCCCCACGCATGGCTT	
NfixinsR	ATCGTCGACAGGACGCAGTGTCCCCTTAAGC	
Pph1insF	ACTGCGGCCGCAGGACGATGTCTGCTCACTCTGCGGT	
Pph1insR	ATCGTCGACAGGACTTTATATCGGTTACATAAATG	
Ldb3insF	ACTGCGGCCGCAGGACGGGCACCCCTATTGAGCATGCT	
Ldb3insR	ATCGTCGACAGGACTCTCGGATTCTCCACGGGACT	
NASPinsF	ACTGCGGCCGCAGGACCAATACCTAGTGTAAC	
NASPinsR	ATCGTCGACAGGACAGCCTACCAAGTTTCA	
CTRLinsF	ACTGCGGCCGCAGGACGGAGCCTTAGATGTGTCACT	
CTRLinsR	ATCGTCGACAGGACTGAGCAGGTGGGTTGCCTT	
Mbnl1F	ACTGCGGCCGCAGGAAACGGACATCAGTGAATGCTACTTAGTAA	
Mbnl1R	ATCGTCGACAGGAAACGGATGTGCTGTCGTCAGTCTAGCTGG	
Mbnl1mtF	CTGGTTTTCCCTTCCTTGTTGTCAGCTAGACT	
Mbnl1mtR	CTGACAACAAGGAAGGGAAAACCAGAGGCTGC	
CTG17F	GGCCGCGGCCC$(CTG)_{17}$GGGCCG	
CTG17R	TCGACGGCCC$(CAG)_{17}$GGGCCGC	
**Splicing analyses**		
**Primer name**	**Sequence 5'→3'**	**Organism**
Atp2A1_F	ATCTTCAAGCTCCGGGCCCT	*Mm*
Atp2A1_R	CAGCTTTGGCTGAAGATGCA	*Mm*
ATP2A1_F	GCTCATGGTCCTCAAGATCTCAC	*Hs*
ATP2A1_R	AGCTCTGCCTGAAGATGTGTCAC	*Hs*
Pphln1_F	ACACAGCAGATCCGGCTCCAGT	*Hs/Mm*
Pphln1_R	AGCCCACTTGCTTTCAGCCTCA	*Hs/Mm*
NASP_F1	GGTGTGCATGTGGAAGAGG	*Hs*
NASP_F2	AGCTGGAGATGGGGTTGATA	*Hs*
NASP_R1	TTTGGCATTTCTTCGGTCTT	*Hs*

NFIX_F	GAGCCCTGTTGATGACGTGTTCTA	*Hs*
NFIX_R	CTGCACAAACTCCTTCAGTGAGTC	*Hs*
Nfix_F	GAGTCCAGTAGATGATGTGTTCTA	*Mm*
Nfix_R	CTGCACAAACTCCTTCAGCGAGTC	*Mm*
LDB3_F	AGCCAGAAGATGAGGCTGACGA	*Hs*
LDB3_R	TCACTGTAGCTGGTGTGGGTGGCA	*Hs*
Ldb3_F	ACCCTGATGAAGAGGCTCTGCG	*Mm*
Ldb3_R	TGGACCCTCGCTGTAGCTGGTA	*Mm*
MBNL1ex1F	CAGCGACATGCAACAGTCTT	*Hs*
MBNL1ex1R	TGTCAGCAGGATGAGCAAAC	*Hs*
Mbnl1ex1F	GAGTCGCGATCCCACAATG	*Mm*
Mbnl1ex1R	TGTCAGCAGGATGAGCAAAC	*Mm*
MBNL2_F	TCCTTTACCAAAGAGACAAGCAC	*Hs*
MBNL2_R	CTCAATGCAGATTCTTGGCATTCC	*Hs*
NCOR2_F	ACACCCACAACCGGAATGAGCCTG	*Hs*
NCOR2_R	GGACTTGGCTTTTCGGCTGCTG	*Hs*
PHKA1_F	TGCACACACTTGAGCTTCATGGA	*Hs*
PHKA1_R	AAAGTCCACCTCCCCAGACTGGTC	*Hs*

Hs, *Homo sapiens*; *Mm*, *Mus musculus*

Supplementary Table S2

Primers' sequences for *in vitro* analysis

**Primer name**	**Sequence 5'→3'**	**Ta [°C]**
Atp2a1_F	GGTGGGATTTTCTCCCAACCT	60
Atp2a1_R	GCTGTATCTCCTTGCGCATC	
Atp2a1_TF	TAATACGACTCACTATAGGGCACCCCTGCGGTCACT	
Atp2a1_TR	AGCAGACTGGACGGCCA	
Atp2a1mt_upF	TAATACGACTCACTATAGGGCACCCCTGCGGTCACTTGCGCGATCTCGCA	
Atp2a1mt_upR	Atp2a1_TR	
Atp2a1mt_#1F	ATGTTGTTGCCACTGCCCGCTTC	
Atp2a1mt_#1R	GGGCAGTGGCAACAACATCAGTTG	
Atp2a1mt_#2F	ACACTGACCTCTTCCACATGAG	
Atp2a1mt_#2R	CATGTGGAAGAGGTCAGTGTCAACA	
Atp2a1mt_up#1F	Atp2a1mt_#1F on Atp2a1_up#1 template	
Atp2a1mt_up#1R	Atp2a1mt_#1R	
Atp2a1mt_#1-#2F	ATGATGTTGACACTGACCTCTTCCACATGA	
Atp2a1mt_#1-#2R	AGGTCAGTGTCAACATCATCAGTGGCTCCAG	
Atp2a1mt_YGCYF	CACATGAGGGCCATGACGTTA	
Atp2a1mt_YGCYR	TAACGTCATGGCCCTCATGTG	
Atp2a1mt_YGCY_TF	TAATACGACTCACTATAGGGCACCCCTGCGGTCACTTGCGCGATCTCGCA	
Pphln1_F	AGGTAGTTGGTTGTCTGGGC	60
Pphln1_R	CTCAGACTTCGCCTTCCAGA	
Pphln1_TF	TAATACGACTCACTATAGGGACGATGTCTGCTCACTCTGCGGT	
Pphln1_TR	GGGACTTTATATCGGTTACATAAATG	
NASP_F	GGTGTGCATGTGGAAGAGG	55

NASP_R	TCTCAACTCTTCCCTTGCTTC	
NASP_TF	TAATACGACTCACTATAGGCAATACCTAGTGTAACAAGC	
NASP_TR	AGCCTACCAAGTTTCAGCAGATGGC	
Nfix_F	CTTTATCGACCGTCAGGAGCA	55
Nfix_R	GCAGTGTCCCCTTAAGCCCC	
Nfix_TF	TAATACGACTCACTATAGGGAGCGGGCTGCTAGCTGGCTTCCT	
Nfix_TR	CCCCGCCCCCATCTCTC	
Ldb3_F	CCAGACTGTCCTTTCCACCC	60
Ldb3_R	ATCCAGCTGTCCACCAATCG	
Ldb3_TF	TAATACGACTCACTATAGGGCACCCCTATTGAGCATGCT	
Ldb3_TR	CTCGGATTCTCCACGGGACT	
Clcn1_F	CCTTCCATGTTTCCTCCTGTG	57
Clcn1_R	ACCAAGGTAGGGAGGAAGTG	
Clcn1_TF	TAATACGACTCACTATAGGCCCCCGTTCTTCTGTG	
Clcn1_TR	AAAGTAGCATCCCACGCCCA	
Tnnt3_F	GAAGGTAGAGACCGGCTTCG	57
Tnnt3_R	GTCACTGAGGAACCATGCCA	
Tnnt3_TF	TAATACGACTCACTATAGGACAGCAAGTATCCATGAAGG	
Tnnt3_TR	GCACAAGCGCACATGCAAGA	
Mbnl1_F	GATGGCTGGCTGCAATATGCC	57
Mbnl1_R	CCTAGGGCAATGGCAGATACTC	
Mbnl1_TF	TAATACGACTCACTATAGGGTTGGTACTAAGAAGTGCCT	
Mbnl1_TR	CCTAGGGCAATGGCAGATACTC	
Mfn-Ctrl1_F	GCAGGCAAGCACACTTACTG	57
Mfn-Ctrl1_R	GCTGGGTGAACCTGAACACT	
Mfn-Ctrl1_TF	TAATACGACTCACTATAGGGACTGGAGAGATGGCTCAGT	
Mfn-Ctrl1_TR	GCTGGGTGAACCTGAACACT	
Mfn-Ctrl3_2F	GCTAGCAACTTATTTGAGGAGCC	55
Mfn-Ctrl3_2R	AGCAGGTGGGTTGCCTT	
Mfn-Ctrl3_2TF	TAATACGACTCACTATAGGAGCCTTAGATGTGTCACT	
Mfn-Ctrl3_2TR	AGCAGGTGGGTTGCCTT	
Atp2a1-Ctrl2_F	TGTTCCCCCTGCTTTGTTCA	57
Atp2a1-Ctrl2 _R	TCTCCATGACGGTCTGTGAC	
Atp2a1-Ctrl2 _TF	TAATACGACTCACTATAGGGCTTCCTGCTGTCATCACC	
Atp2a1-Ctrl2_TR	CTGACATCTGGTTGGTGGTG	
Capzb-Ctrl4_F	CCTTCTCTCTGCGGTCGGTC	57
Capzb-Ctrl4_R	CTCCCCAGAAAGGGGCAAAC	
Capzb-Ctrl4_TF	TAATACGACTCACTATAGGGACTTCTCACGACTTAGCCT	
Capzb-Ctrl4_TR	CCAGAGGCTGCCCAGAAAG	
SRSF1_F	ATCGGGATCCAATGTCGGGAGGTGGTGTGATTCGT	
SRSF1_R	AACTCGGCGGCCGCTCATACCTCATGAGATCTAAACTTAGTG	

Supplementary Table S3

Sequences of DNA antisense oligonucleotides

AONs' name	Sequence 5'→3'
DNA	GCGGGCAGTGGCAACAGCAGC
DNA-up	GCTGCGAGCGCGCGCAAGTGACCGC
DNA-dw	AGTAACGGCATGGCCCTCAT
DNA-Pphln1	GTGGAGAAGCAAAAAGCAAGA
DNA-Pphln1-2	AGCAAAAACCGCAGAGTGAGCA
DNA-NASP	AGCATCCCGGCTAGCAGAGCA
DNA-NASP-2	AGCCTACCAAGTTTCAGCAGATGGC
DNA-Nfix	CCAGCAAGCACAGGCAGCGGG
DNA-Nfix-2	GCAGGGCCTGGGGGGAGGAAGCAG
DNA-Ldb3	GGCAGAAGCAGGCAGCAGCG
DNA-Ldb3-2	CTGGTGCACACTGGAGCATG
DNA-Ldb3-3	CGAGGCCGCAATGGGAGAGGCAGCAG
DNA-Ldb3-4	CGGCAGCAGCATGGGTGGCAGCAG
DNA-Ldb3-5	GCGGCAGGGCCTGCAGCA

Supplementary Table S4

Coordinates of analyzed alternative exons in human (hg19) and mouse (mm10) genomes

gene name & alternative exon	human genome coordinates	mouse genome coordinates
ATP2A1 ex22	hg19 chr16:28,915,020-28,915,064	mm10 chr7:126,446,540-126,446,594
PPHLN1 ex6	hg19 chr12:42,384,939-42,384,997	mm10 chr15:93,452,117-93,452,174
NASP ex7	hg19 chr1:45,607,312-45,608,363	not analyzed
NFIX ex7	hg19 chr19:13,078,607-13,078,736	mm10 chr8:84,723,727-84,723,851
LDB3 ex10	hg19 chr10:88,466,287-88,466,478	mm10 chr14:34,555,340-34,555,529
MBNL1 ex1	hg19 chr3:152,299,398-152,300,377	mm10 chr3:60,528,755-60,529,811
MBNL2 ex7	hg19 chr13:97,365,135-97,365,172	not analyzed
NCOR2 ex45	hg19 chr12:124,327,386-124,327,638	not analyzed
PHKA1 ex19	hg19 chrX:72,620,713-72,620,927	not analyzed

Supplementary Figures

Supplementary Figure S1

a

Fragments of transcripts of previously confirmed MBNL1 targets:

Atp2a1:
GGGCACCCCUGCGGUCACUUGCGCGCGCUCGCAGCUCUCCCUGGAGCC**ACUGCU**GCUGUUGCCACUGCC**CGCU**UCC
ACAUGAGGGCCAUGCCGUUACUGUGGGCACUGGCAGCUGUGAGGCUGUGUCCUGGCCGUCCAGUCUGCUA

Clcn1:
GGCCCCCGUUCUUCUGUGCUUCCUGACACCCAUCCACCUGGUUUACAUACCACCUGUCUGUCCCCCUCUGCCACCUG
CCUCGCCCGUCGUGCUUCUCUGUUGCAGACCGUGCCUGGGCAGCUUGAUCUCCUGGUGCCAGCCUGUGCAGUGGGC
GUGGGAUGCUACUUUA

Tnnt3:
GGACAGCAAGUAUCCAUGAAGGGGAGGAGACCUAUGGGAAGGUGUAGGAGCUGCCCUGCCACCCUCAGGCAGGGGG
CUCUGUGCGUGCCUGCCUCUGCUGUUCCAUGUGGCCACUGCUGCUGUGUGGCCUCUGCACUUCUGCAGCUGCGUGG
CUUUUUUCUUGCAUGUGCGCUUGUGC

Fragments of transcripts - negative controls:

***Mfn*-Ctrl1:**
GGGACUGGAGAGAUGGCUCAGUGGUUAAGAGCGCUGGCUGCUCUUGCAGAGUCCAUAAGUUCAGUUCCCAGCUCAUC
AUCAUCUGUAGUUGGAUCUGAUGCUCUCUUCCAGUGUGCCAUAAAUAAAAUUUUUUUUUAAAGUACUGAAGAGUCAGU
GUUCAGGUUCACCCAGCA

***Atp2a1*-Ctrl2**:
GGGCUUCCUGCUGUCAUCACCACCUGCUUGGCUUUGGGUACCCGCCGGAUGGCAAAGAAGAACGCCAUCGUGAGGAG
UCUGCCCUCUGUGGAGACCCUGGGCUGUACCUCUGUCAUCUGUUCUGACAAAACAGGGACCCUCACCACCAACCAGA
UGUCAGA

***Mfn*-Ctrl3:**
GGCACCUAUCAGUGACCUUUUUCCUUCUACCAAGUCACUUGUGGGCUGGGUACUACUUAUCUCCUCCAGCUGACCUG
UUUAUUCUGUUUCCAUGGUAGCAGGUGUAAGGCAUGCAGGAUGUUAGUAGGGUUACUAACCAACAUUGCA

***Capzb*-Ctrl4:**
GGGAGCCUUAGAUGUGUCACUUCCCUCCUCCCCAUACCCCUGCACCCCCUAAUCUACCUGGAUACCUGCAGAGCAG
GCCCACCAUCUUUGGUGGUGGAAGGGCCAGAGAACUACCUCACAUGGUCAAGGAAGCCUUGUAAGGUAGGCUGAGAG
GGAAGGCAACCCACCUG

b

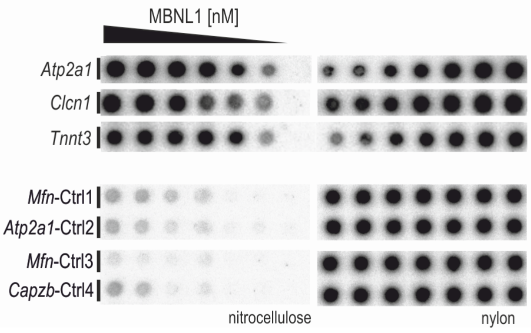

c

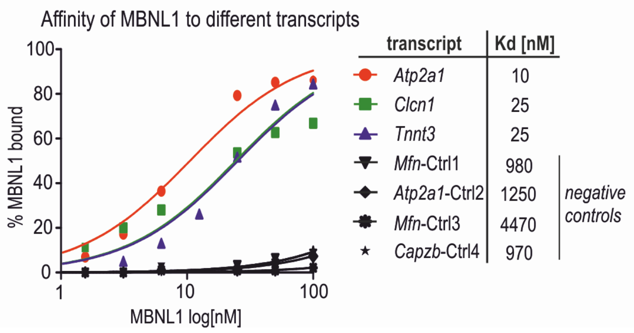

Supplementary Figure S1

The presence of YGCY motifs is not sufficient for MBNL1 binding in *in vitro* tests. (**a**) The sequences of transcript fragments previously described in literature as MBNL1 bound: *Atp2a1*[1], *Clcn1*[2], *Tnnt3*[3] and ~150 nt control RNAs (RNA-Ctrl) with or without YGCY motifs in their sequence. YGCY motifs are marked with green and in bold if conservatively maintained. (**b**) Filter binding assay (FBA) represents the affinity of recombinant MBNL1 to particular transcripts. Nitrocellulose membrane holds signals derived from the MBNL1/RNA complexes, whereas the nylon membrane keeps signals of free RNA. MBNL1 concentration used for *Atp2a1*, *Clcn1*, *Mfn*-Ctrl1, *Atp2a1*-Ctrl2, *Mfn*-Ctrl3, *Capzb*-Ctrl4: 100, 50, 25, 6.25, 3.1, 1.5, 0 nM and for *Tnnt3*: 100, 50, 25, 12.5, 6.25, 3.1, 0 nM; the data coming from different parts of the same membrane are divided with white space; n = 1 (**c**) The quantification of FBA results. Dissociation constant (Kd) for each previously examined fragment of transcript oscillates between 10-25 nM, whereas for control samples the Kd value is at least 38 fold higher.

Supplementary Figure S2

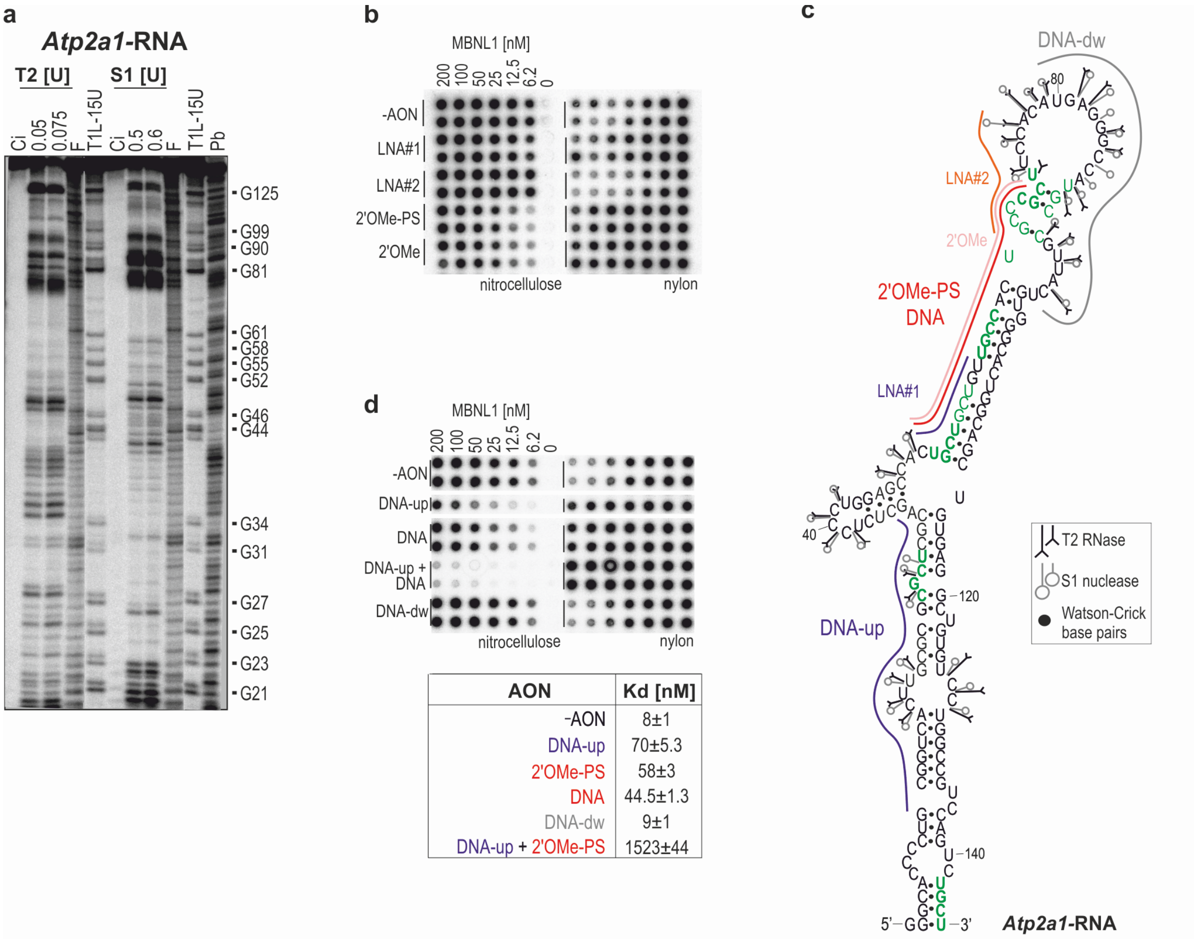

AON	Kd [nM]
-AON	8±1
DNA-up	70±5.3
2'OMe-PS	58±3
DNA	44.5±1.3
DNA-dw	9±1
DNA-up + 2'OMe-PS	1523±44

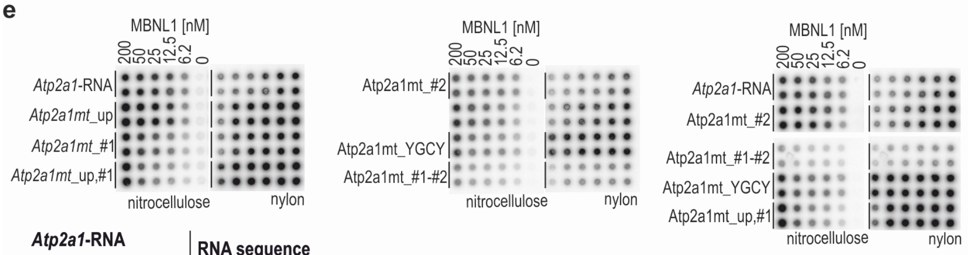

Atp2a1-RNA mutants	RNA sequence (up, #1, #2, dw)	Kd [nM]
Atp2a1-RNA	...CGCGCUCGCAGCUCUCCCUGGAGCCACUGCUGCUGUUGCCACUGCCCGCUUCCACAUGAGGGCCAUGCCGUU...	8±2
*Atp2a1*mt_up	...CGAUCUCGCAGCUCUCCCUGGAGCCACUGCUGCUGUUGCCACUGCCCGCUUCCACAUGAGGGCCAUGCCGUU...	15±2
*Atp2a1*mt_#1	...CGCGCUCGCAGCUCUCCCUGGAGCCACUGAUGUUGUUGCCACUGCCCGCUUCCACAUGAGGGCCAUGCCGUU...	25±2
*Atp2a1*mt_up,#1	...CGAUCUCGCAGCUCUCCCUGGAGCCACUGAUGUUGUUGCCACUGCCCGCUUCCACAUGAGGGCCAUGCCGUU...	56±16
*Atp2a1*mt_#1-#2	...CGCGCUCGCAGCUCUCCCUGGAGCCACUGAUGUUGUUGACACUGACCUCUUCCACAUGAGGGCCAUGCCGUU...	24±3
*Atp2a1*mt_#2	...CGCGCUCGCAGCUCUCCCUGGAGCCACUGCUGCUGUUGACACUGACCUCUUCCACAUGAGGGCCAUGCCGUU...	10±2
*Atp2a1*mt_YGCY	...CGAUCUCGCAGCUCUCCCUGGAGCCACUGAUGUUGUUGACACUGACCUCUUCCACAUGAGGGCCAUGACGUU...	65±16

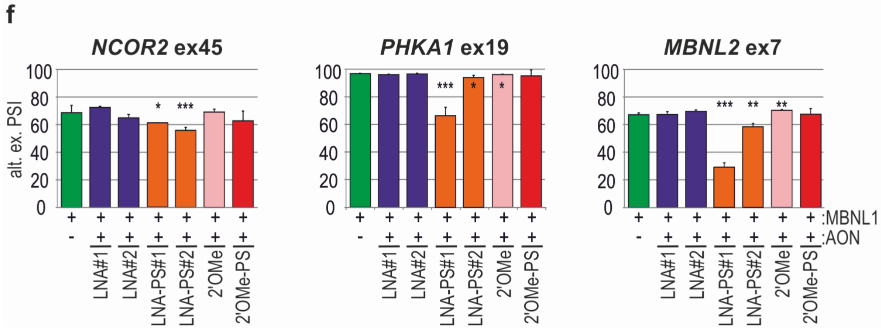

Supplementary Figure S2

Indication of significant MBNL1-binding motifs within *Atp2a1*-RNA *via* DNA-AONs and mutagenesis. (**a**) The structure probing of analyzed 5'-^{32}P-labeled *Atp2a1*-RNA fragment using RNases. Ci- untreated RNA samples; F – formamid ladder; Pb – lead ion ladder; T1L - G ladder formed by RNase T1 cleavage in denaturing conditions; T2 and S1 - endonucleases which cleave single stranded regions; particular guanosines are marked as G on the right side of the picture. The cleavage sites, and intensities for the selected probes are shown by using symbols explained in the inset. (**b**) Representative raw data from FBA showing differently modified AON's inhibitory properties of MBNL1/RNA complexes. (**c**) As in **Figure 1c** but with additional DNA-AONs bound *in vitro* to regions upstream (DNA-up, marked with dark blue) and downstream (DNA-dw, marked with grey) of major MBNL1 binding regions #1 and #2. (**d**) *Top*, representative raw data from FBA showing the blocking properties of AONs (DNA-based) and revealing additional MBNL1 bound region upstream region #1; the data coming from different parts of the same membrane are divided with white space. *Bottom,* quantification of FBA. The Kd values are presented with SD; n = 2. (**e**) Top, representative raw data from FBA showing reduced affinity of MBNL1 to *Atp2a1*-RNA mutants of significant binding sites; the data coming from different parts of the same membrane are divided with white space. *Bottom*, the sequences of Atp2a1-RNA mutants; YGCY motifs are marked with green and in bold if conservatively maintained. The major MBNL1-binding regions are marked as #1 and #2; up, the YGCY motif upstream from region #1; dw, the YGCY motif downstream from region #2. On the right, quantification of FBA. The Kd values are presented with SD; n = 4. (**f**) Percentage of MBNL-dependent alternative exons inclusion in *NCOR2*, *PHKA1* and *MBNL2* transcripts upon MBNL1 overexpression and treatment with 100 nM of *Atp2a1*-specific AONs; n = 3.

Supplementary Figure S3

a

***Pphln1*-RNA**

DNA-2 | DNA / 2'OMe-PS

GGGACGATGTCTGCTCACTCTGCGGTTTTTGCTGTCATCTGTGTTTATGTCTTTGTCTCTTGCTTTTTGCTTCTCCACTCATTTATGTAACCGATATAAAGTC

CC

***NASP*-RNA**

DNA / 2'OMe-PS

GGCAATACCTAGTGTAACAAGCTTGGGTTTCTTTTGCAGTAAGTTGCTCTGCTAGCCGGGATGCTCCCTTTCCTTTTCTCTAGGGGGCAGATAATTTTTAG

DNA-2

GCCATCTGCTGAAACTTGGTAGGCT

***Nfix*-RNA**

DNA-2

GGGAGCGGGCTGCTTAGCTGGCTTCCTACCCCAGCCAGCTAAACCTGCGTGTTGCTGCTTCCTCCCCCCAGGCCCTGCTTCTCTAAAGAAGTCAGGAAA

ex7

DNA / 2'OMe-PS

GCTGGACTTCTGCAGCGCCCTCTCCTCTCAGGGCAGTTCCCCACGCATGGCTTTCACCCACCACCCGCTGCCTGTGCTTGCTGGAGTCAGACCAGGTG

ex7

AGAGATGGGGGCGGGG

***Ldb3*-RNA**

DNA-2 | DNA / 2'OMe-PS | DNA-3

GGCACCCCTATTGAGCATGCTCCAGTGTGCACCAGCCAGGCCACTTCCCCGCTGCTGCCTGCTTCTGCCCAGTCGCCCGCTGCTGCCTCTCCCATTGC

DNA-4 | DNA-5

GGCCTCGCCAACCCTGGCCACAGCTGCTGCCACCCATGCTGCTGCCGCCTCTGCTGCAGGCCCTGCCGCAAGTCCCGTGGAGAATCCGAG

b

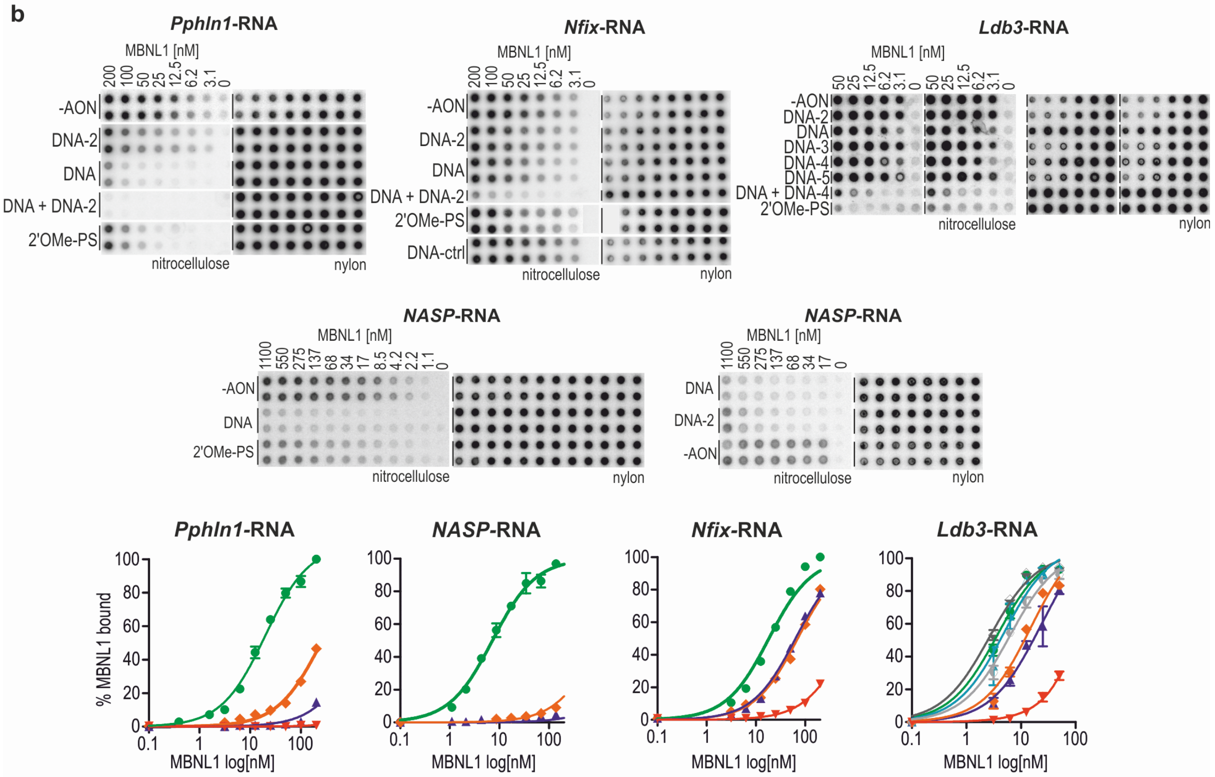

***Pphln1*-RNA**

AON	Kd [x] (fold change)
-AON	1
DNA	92x
DNA-2	15x
DNA + DNA-2	1357x

***NASP*-RNA**

AON	Kd [x] (fold change)
-AON	1
DNA	980x
DNA-2	145x

***Nfix*-RNA**

AON	Kd [x] (fold change)
-AON	1
DNA	3x
DNA-2	4x
DNA + DNA-2	42x

***Ldb3*-RNA**

AON	Kd [x] (fold change)
-AON	1
DNA	5x
DNA-4	4x
DNA + DNA-4	45x
DNA-2	0.8x
DNA-3	1x
DNA-5	2x

Supplementary Figure S3

DNA-AONs sufficiently block MBNL1 binding motifs *in vitro*. (**a**) The sequences of *Atp2a1*, *Pphln1*, *NASP*, *Nfix*, *Ldb3* and *Mbnl1* transcript fragments with highlighted YGCY containing regions bound by specific DNA-AONs or RNA-based AONs *in vitro*. (**b**) *Top*, representative raw data from FBA showing the blocking properties of AONs (DNA- and RNA-based) and indicating significant MBNL1-binding regions; the data coming from different parts of the same membrane are divided with white space. *Bottom*, quantification of FBA; below each chart, there is a fold change of Kd values normalized to Kd for MBNL1/RNA complexes without AON application (-AON); n = 2.

Supplementary Figure S4

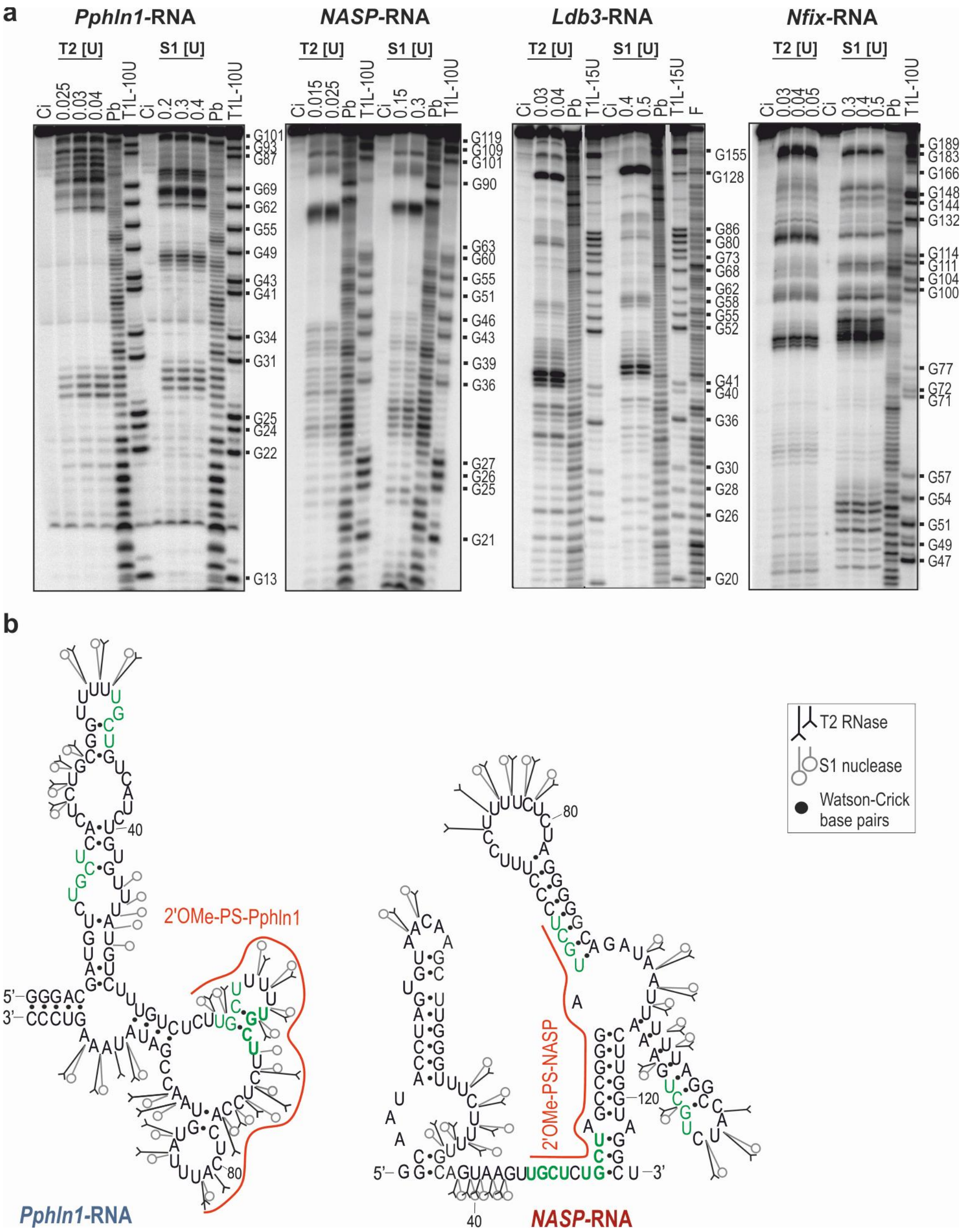

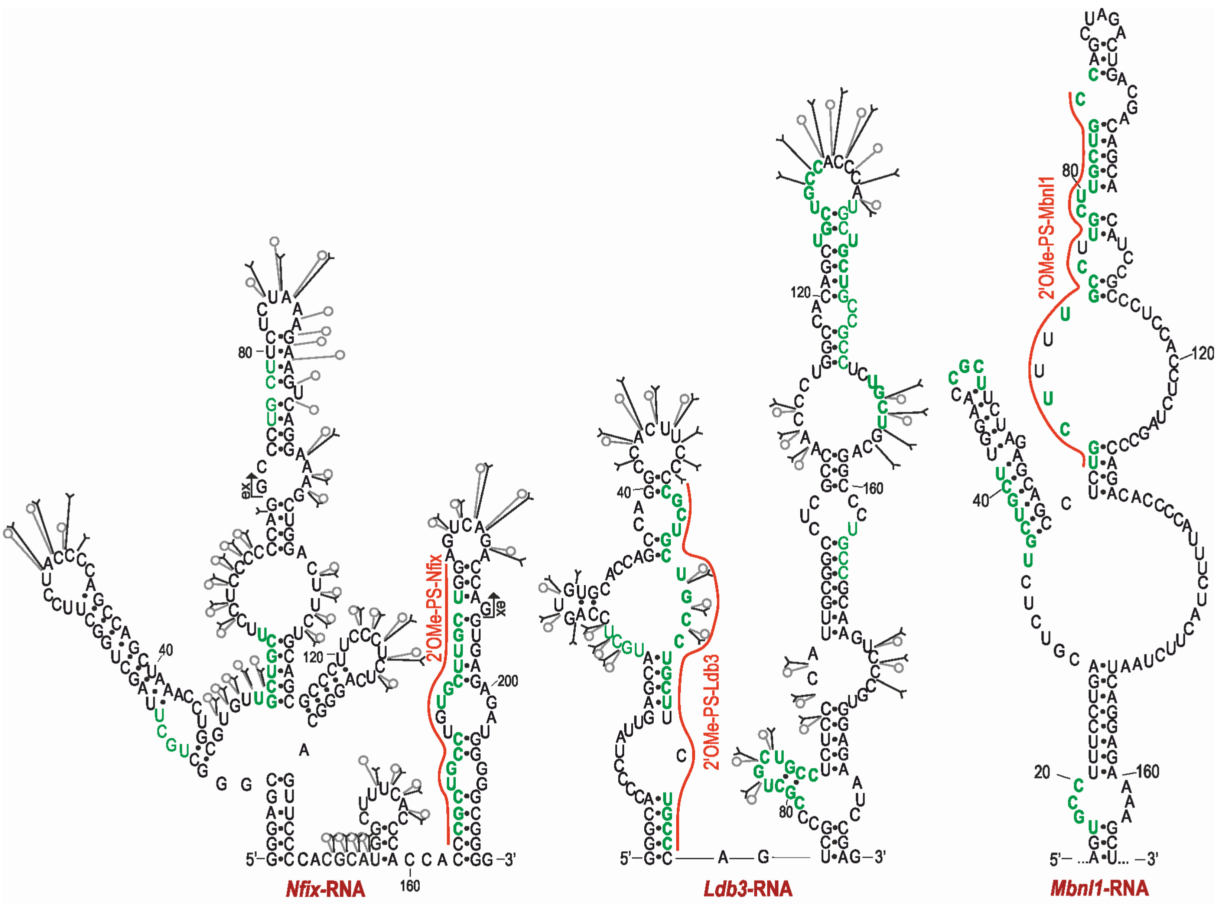

c

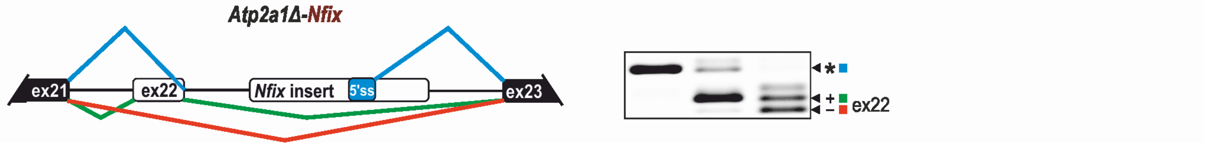

Enzymatic probing of short intronic and exonic RNA fragments. (**a**) As in **Supplementary Figure S2a** but for 5'-^{32}P-labeled *Pphln1-*, *NASP-*, *Nfix-*, *Ldb3*-RNA using specific RNases. Ci-untreated RNA samples, Pb – lead ion ladder, F – formamid ladder, T1L - G ladder formed by RNase T1 cleavage in denaturing conditions; particular guanosines are marked as G on the right of each electrophoregram. (**b**) Experimentally determined secondary structures of short intronic and exonic RNA fragments containing YGCY motifs marked with green and in bold if present in human and mouse. Significant for MBNL1-binding regions and complementary to particular AONs are indicated with a red line. (**c**) A schematic representation of splicing isoforms' distribution of *Atp2a1Δ-Nfix* mRNA*.* An asterisk points to an artificial isoform composed of *Nfix* insert and *Atp2a1* intron 22 fragment.

Supplementary Figure S5

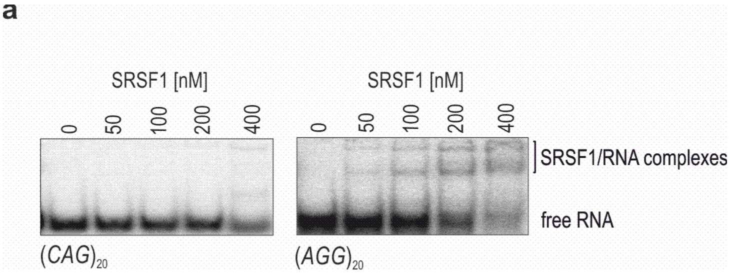

Specificity of SRSF1 binding *in vitro*. (**a**) EMSA on a native PA gel showing no or weak interaction between 5'-^{32}P-labeled negative control RNA sequences (*CAG*)$_{20}$ and (*AGG*)$_{20}$ and SRSF1 at indicated concentrations.

Supplementary Figure S6

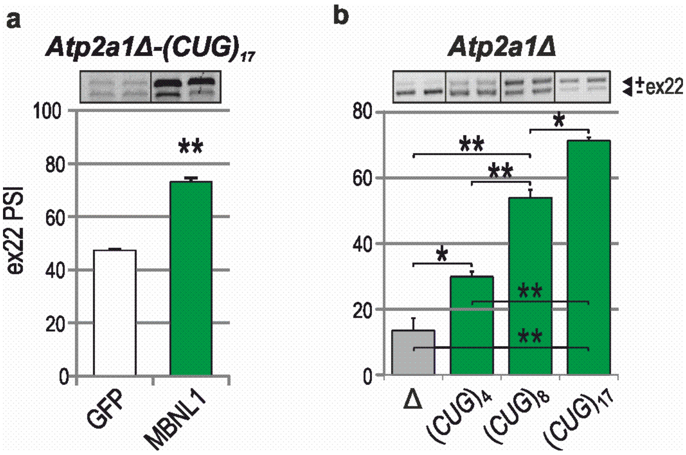

The hybrid minigene is useful for screening of potential RNA binding therapeutics. (**a**) The efficacy of alternative ex22 inclusion into *Atp2a1-(CUG)$_{17}$* mRNA induced by endogenous MBNLs (white bar) or MBNL1 overexpression (green bar); n = 2. (**b**) A gradual increase in the number of MBNL1-binding sites in *Atp2a1Δ-(CUG)n* (n = 4, 8, 17) minigenes positively correlates with the enhancement of alternative ex22 inclusion upon MBNL1 overexpression; n = 2.

Supplementary Figure S7

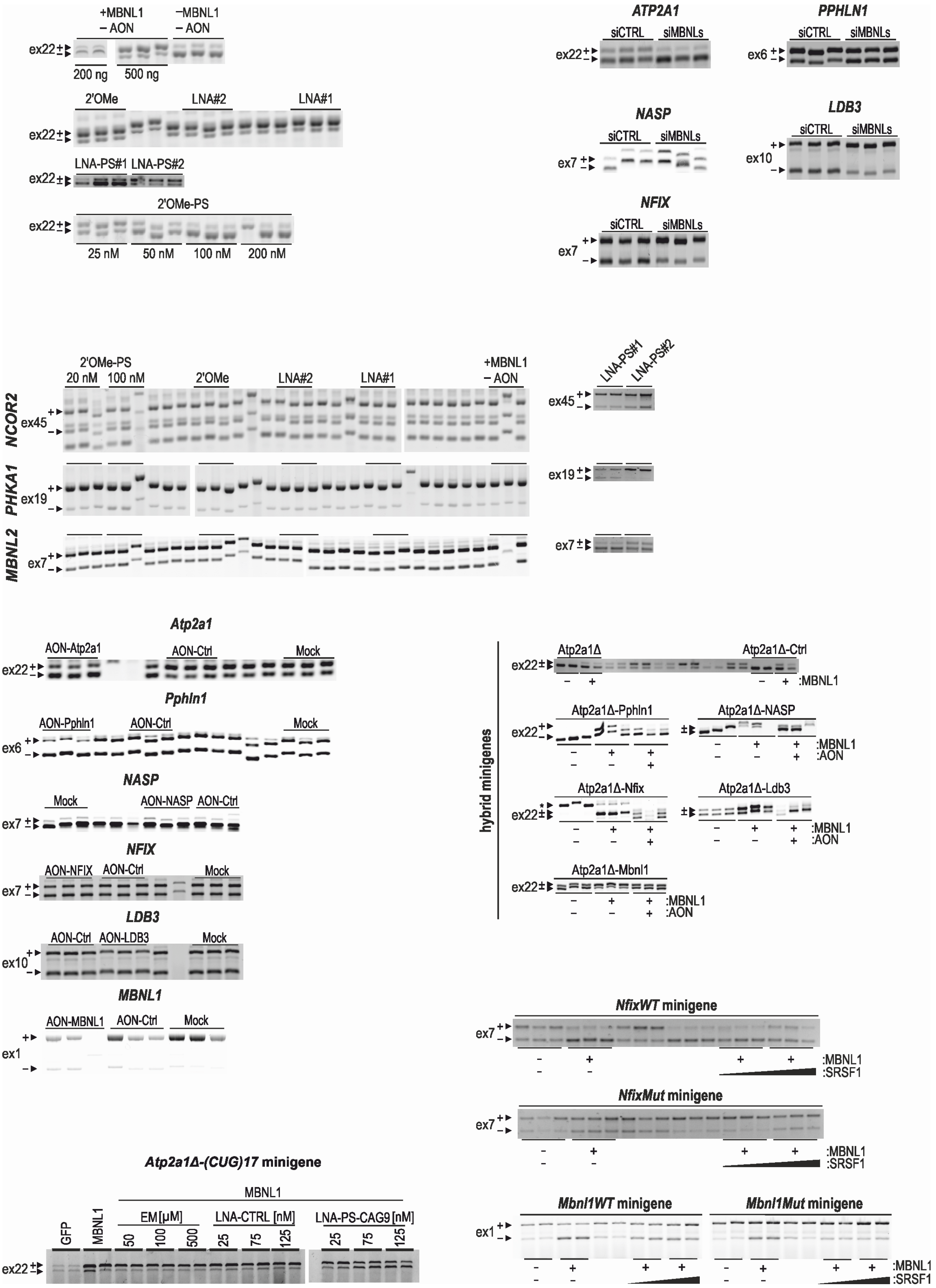

Supplementary Figure S7

Raw images of RT-PCR analyses of alternative splicing. (**a**) Original agarose gels presenting all RT-PCR results of alternative splicing analyses on which calculations and statistical data were based.

Supplementary references

1. Hino, S. *et al.* Molecular mechanisms responsible for aberrant splicing of SERCA1 in myotonic dystrophy type 1. *Hum Mol Genet* **16**, 2834-2843, doi:10.1093/hmg/ddm239 (2007).
2. Kino, Y. *et al.* MBNL and CELF proteins regulate alternative splicing of the skeletal muscle chloride channel CLCN1. *Nucleic Acids Res* **37**, 6477-6490, doi:10.1093/nar/gkp681 (2009).
3. Yuan, Y. *et al.* Muscleblind-like 1 interacts with RNA hairpins in splicing target and pathogenic RNAs. *Nucleic Acids Res* **35**, 5474-5486, doi:10.1093/nar/gkm601 (2007).
